# Protein arginine methyltransferase 3-induced metabolic reprogramming is a vulnerable target of pancreatic cancer

**DOI:** 10.1186/s13045-019-0769-7

**Published:** 2019-07-19

**Authors:** Ming-Chuan Hsu, Ya-Li Tsai, Chia-Hsien Lin, Mei-Ren Pan, Yan-Shen Shan, Tsung-Yen Cheng, Skye Hung-Chun Cheng, Li-Tzong Chen, Wen-Chun Hung

**Affiliations:** 1 National Institute of Cancer Research, National Health Research Institutes, No. 367, Shengli Road, Tainan, 704 Taiwan; 2 Institute of Clinical Medicine, College of Medicine, Kaohsiung Medical University, Kaohsiung, 807 Taiwan; 3 Institute of Clinical Medicine, National Cheng Kung University, Tainan, 704 Taiwan; 4 Department of Surgery, National Cheng Kung University Hospital, Tainan, 704 Taiwan; 5 Department of Surgery, Koo Foundation Sun Yat-Sen Cancer Center, Taipei, 112 Taiwan; 6 Department of Radiation Oncology, Koo Foundation Sun Yat-Sen Cancer Center, Taipei, 112 Taiwan; 7 Division of Hematology/Oncology, Department of Internal Medicine, National Cheng Kung University Hospital, Tainan, 704 Taiwan; 8 Graduate Institute of Medicine, College of Medicine, Kaohsiung Medical University, Kaohsiung, 807 Taiwan

**Keywords:** Protein arginine methyltransferase 3, Methylation, Glyceraldehyde-3-phosphate dehydrogenase, Metabolic reprogramming

## Abstract

**Background:**

The biological function of protein arginine methyltransferase 3 (PRMT3) is not well known because very few physiological substrates of this methyltransferase have been identified to date.

**Methods:**

The clinical significance of PRMT3 in pancreatic cancer was studied by database analysis. The PRMT3 protein level of human pancreatic tumors was detected by immunoblotting and immunohistochemical staining. PRMT3-associated proteins and the methylation sites on the proteins were investigated using mass spectrometry. Seahorse Bioscience analyzed the metabolic reprogramming. Combination index analysis and xenograft animal model were conducted to explore the effects of combination of inhibitors of glyceraldehyde-3-phosphate dehydrogenase (GAPDH) and oxidative phosphorylation on tumor growth.

**Results:**

We found that the expression of PRMT3 is upregulated in pancreatic cancer, and its expression is associated with poor survival. We identified GAPDH as a PRMT3-binding protein and demonstrated that GAPDH is methylated at R248 by PRMT3 in vivo. The methylation of GAPDH by PRMT3 enhanced its catalytic activity while the mutation of R248 abolished the effect. In cells, PRMT3 overexpression triggered metabolic reprogramming and enhanced glycolysis and mitochondrial respiration simultaneously in a GAPDH-dependent manner. PRMT3-overexpressing cancer cells were addicted to GAPDH-mediated metabolism and sensitive to the inhibition of GAPDH and mitochondrial respiration. The combination of inhibitors of GAPDH and oxidative phosphorylation induced a synergistic inhibition on cellular growth in vitro and in vivo.

**Conclusion:**

Our results suggest that PRMT3 mediates metabolic reprogramming and cellular proliferation through methylating R248 of GAPDH, and double blockade of GAPDH and mitochondrial respiration could be a novel strategy for the treatment of PRMT3-overexpressing pancreatic cancer.

**Electronic supplementary material:**

The online version of this article (10.1186/s13045-019-0769-7) contains supplementary material, which is available to authorized users.

## Background

The methylation of arginine residues in cellular proteins by protein arginine methyltransferases (PRMTs) is an important posttranslational modification that modulates diverse cellular processes including gene transcription, DNA repair, messenger RNA processing, and signal transduction [1, 2]. PRMTs introduce monomethylation as well as symmetric or asymmetric dimethylation on their substrates by using *S*-adenosyl-l-methionine (SAM) as the methyl donor. Among the nine identified PRMTs in mammalian cells, PRMT3 is unique in several ways. First, PRMT3 contains a C2H2 zinc finger domain that is not presented in other PRMTs and this domain is crucial for substrate recognition [3]. Second, PRMT3 is localized predominantly (or exclusively) in the cytoplasm under physiological circumstances, while other PRMTs are distributed both in the nucleus and cytoplasm or shuttled between these two compartments [3–5]. Although PRMT8 has also been suggested to be a cytosolic protein and may be recruited to the plasma membrane via myristoylation-mediated attachment, subsequent studies demonstrated that it is predominantly found in the nuclei of neuronal cells [6, 7]. Third, no histone proteins have been found to be methylated by PRMT3 in vivo until now. The existence of PRMTs in the nucleus suggests the possibility that these enzymes may directly methylate histone proteins to regulate gene expression via epigenetic modification. For instance, the methylation of histone H4 at arginine 3 (H4R3) is frequently detected in eukaryotic cells and this methylation is mainly catalyzed by PRMT1 [8]. Another histone marker H3R17 has been shown to be methylated by PRMT4, and the methylation plays a critical role in the induction of class II major histocompatibility genes by interferon-γ [9]. A recent study demonstrated that PRMT6 methylates H3R2 to induce a global DNA hypomethylation by attenuating the recruitment of DNA methyltransferase 1 accessary factor UHRF1 to histone H3 [10]. To date, no arginine residues of histone proteins have been shown to be specifically methylated by PRMT3 in vivo.

The biological function of PRMT3 remains elusive due to the limited physiological substrates identified. Two previous studies demonstrated that the 40S ribosomal protein S2 (rpS2) is an in vivo PRMT3 substrate [11, 12]. The results showed that PRMT3 interacted with rpS2 via the zinc finger domain and methylated rpS2 in vitro. Interestingly, the 40S:60S free ribosomal subunit ratio was changed while the processing of pre-ribosomal RNA was largely unaffected in PRMT3-depleted cells. The knockout of PRMT3 in mice did not influence viability, although the animal size was smaller [13]. The methylation of rpS2 in PRMT3-deficient mice is indeed dramatically reduced suggesting that rpS2 is a physiological substrate of PRMT3. Additional reported PRMT3 substrates include Src-associated substrate during mitosis 68Kd (Sam68), poly(A)-binding protein 1 (PABP1), PABP2, nuclear poly(A)-binding protein (PABPN1), high-mobility group A1, and p53 [14–18]. However, methylation of these proteins by PRMT3 was mainly demonstrated in vitro and the biological consequences induced by methylation in vivo were largely uncharacterized. By using gain-of-function mutant PRMT3 and modified SAM analogs as tools, a recent study identified 83 potential PRMT3 substrates in HEK293T cells [19]. Those substrates are known to be involved in the regulation of various cellular pathways, and four proteins including tubulin alpha-1C chain (TUBA1C), TUBB4A, triosephosphate isomerase (TPI), and keratin type II cytoskeletal 6A (KRT6A) were further validated as PRMT3 substrates by biochemical approaches. However, the role of these substrates in PRMT3-mediated biological effects remains unclear.

In this study, we show that PRMT3 is upregulated in pancreatic cancer and is associated with poor patient survival, suggesting a novel oncogenic function of PRMT3. Moreover, we identified a total of 293 PRMT3-interacting proteins in pancreatic cancer cells and found that PRMT3 methylated GAPDH at arginine 248 to promote glycolysis and mitochondrial respiration simultaneously in cancer cells. The combination of inhibitors of GAPDH and oxidative phosphorylation significantly suppresses cell proliferation in vitro and tumor growth in vivo.

## Materials and methods

### Antibodies, chemicals, and plasmids

Antibodies used were as follows: α-GFP (Abcam #ab290, Cambridge, UK), α-GFP Sepharose (Abcam #ab69314), α-PRMT3 (GeneTex #GTX23765, Irvine, CA, USA), α-asymmetrical dimethyl arginine (α-ADMA) (Cell Signaling Technology #13522, Denvor, MA, USA), α-GAPDH (GeneTex #GTX100118), α-Flag (Sigma, #F1804, St Louis, MO, USA), and α-Actin (Millipore #MAB1501, Birlington, MA, USA). Chemicals were as follows: SGC707 (Cayman #17017, Ann Arbor, MI, USA), cycloheximide (Sigma #C7698), heptelidic acid (BioVision #2215-250, Milpitas, CA, USA), and oligomycin A (Cayman #11342). Plasmids were as follows: The pEGFP-PRMT3 expression vector was kindly provided by Dr. Mien-Chie Hung [20]. pcDNA3-PRMT3 expression vector was a gift from Dr. Jian Jin. Human GAPDH cDNA ORF Clone was purchased from Sino Biological (#HG10094-NF, Beijing, China). R248K-GAPDH mutant was generated using a QuickChange site-directed mutagenesis kit according to the manufacturer’s protocol (Agilent Technologies #200519, Santa Clara, CA, USA). The primers used for mutagenesis are shown as follows (5´–3´):

- F: GTGGTGGACCTGACCTGCAAGCTAGAAAAACCTGCC
- R: GGCAGGTTTTTCTAGCTTGCAGGTCAGGTCCACCAC

### Cell culture and stable cell lines

PANC-1 and HEK293T cells were cultured in DMEM medium with 10% fetal bovine serum (FBS) and 1% penicillin/streptomycin. PANC-1 cells with stable expressions of GFP and GFP-PRMT3 were generated in our lab and maintained in the DMEM medium supplemented with 800 μg/ml G418. GFP/wild-type GAPDH, GFP-PRMT3/wild-type GAPDH, or GFP-PRMT3/R248K-GAPDH mutant co-expressing PANC-1 stable cells was established in our lab and maintained in DMEM medium containing 800 μg/ml of G418 and 200 μg/ml hygromycin B. HPDE cells were kindly provided by Dr. Wun-Shaing Wayne Chang (National Institute of Cancer Research, National Health Research Institutes). HPDE cells were grown in keratinocyte serum-free media (Invitrogen, #17005-042, Carlsbad, CA, USA) supplemented with bovine pituitary extract (25 mg), EGF (2.5 μg), and 1% penicillin/streptomycin. BxPC3 cells were kindly provided by Dr. Kuang-Hung Cheng [21]. BxPC3 cells were cultured in RPMI 1640 medium containing 2 mM glutamine, 10% FBS, and 1% penicillin/streptomycin. Miapaca-2 cells were grown in in DMEM medium with 10% FBS, 2.5% horse serum, and 1% penicillin/streptomycin. Capan-2 cells were a gift from Dr. Wun-Shaing Wayne Chang and maintained in McCoy’s 5a medium supplemented with 10% FBS and 1% penicillin/streptomycin. L3.6pl cells were kindly provided by Dr. Mien-Chie Hung [22]. L3.6pl cells were cultured in DMEM/F12 medium containing 10% FBS and 1% penicillin/streptomycin. Cell line identities were verified by short tandem repeat analysis and were confirmed as *Mycoplasma* free.

### Patient tumor tissue samples and immunoblotting

Human pancreatic tumor tissues were obtained from patients undergoing surgical resection at Koo Foundation Sun Yat-Sen Cancer Center (Taipei, Taiwan) and National Cheng Kung University Hospital (Tainan, Taiwan) under the guidelines approved by the Institution Review Board at National Health Research Institutes. Written informed consent was obtained from each patient. Total proteins were extracted from human pancreatic tumor tissues using AllPrep DNA/RNA/Protein mini kits (Qiagen #80004, Hilden, Germany) following the manufacturer’s instructions. Briefly, tissues were lysed and homogenized in buffer RLT by using TissueRuptor. The lysates were centrifuged at 13,000 rpm for 3 min, and the supernatant was passed through an AllPrep DNA spin column, which allows the binding of genomic DNA. Ethanol was added to the flow-through from the AllPrep DNA spin column, and the mixture was subsequently passed through an RNeasy spin column to collect total RNA. The supplied aqueous protein precipitation solution, buffer APP, was added into the flow-through of RNeasy spin column and incubated at room temperature for 10 min, followed by centrifugation at 13,000 rpm for 10 min. The precipitated protein pellets were resuspended by 500 μl of 70% ethanol and were centrifuged at 13,000 rpm for 1 min. The total proteins were resuspended in 50–100 μl buffer ALO, and equal amounts of proteins were subjected to western blot as described previously [23].

### Immunohistochemical (IHC) staining

Human PDAC tissues were obtained from patients with surgical resection in National Cheng Kung University Hospital (Tainan, Taiwan) under the guidelines approved by the Institutional Review Board of National Cheng Kung University Hospital. Tissue sections were stained with PRMT3 (GeneTex #GTX23765) antibody overnight at 4 °C followed by incubation with horseradish peroxidase (HRP)-conjugated secondary antibodies for 1 h at room temperature. The protein signal was developed using a 3,3′-diaminobenzidine solution.

### Mass spectrometry analysis

GFP-PRMT3 proteins were purified from GFP-PRMT3-overexpressing PANC-1 cells by immunoprecipitation with GFP antibody. The immunoprecipitated complexes were subjected to in-solution digestion with trypsin, and the PRMT3-interacting proteins were identified by mass spectrometry (Mithra Biotechnology Inc., Taiwan). To identify the arginine residue on GAPDH methylated by PRMT3, endogenous GAPDH proteins were purified from GFP-PRMT3-overexpressing PANC-1 cells by immunoprecipitation with GAPDH antibody and the immunoprecipitated complexes were separated by SDS-PAGE. The protein bands corresponding to GAPDH were excised and subjected to in-gel digestion with trypsin. The samples were reduced in 50 mM dithiothreitol at 37 °C for 1 h. Alkylation was conducted using 100 mM iodoacetamide for 30 min in dark at room temperature. The resulting proteins were digested with trypsin at 37 °C overnight. After digestion, the protein fragments were extracted with 10% formic acid and analyzed by liquid chromatography/tandem mass spectrometry (Mithra Biotechnology Inc., Taiwan).

### Metabolite extraction and metabolome analysis

The cells were washed twice by using 5% mannitol solution and were then incubated with 800 μl of methanol at room temperature to inactivate enzymes. The cell extracts were mixed with 550 μl of Milli-Q water containing internal standard solution (Human Metabolome Technologies (HMT), H3304-1002) and incubated at room temperature for 30 s. The extracted solutions were transferred into microtubes and centrifuged at 2300×*g*, 4^o^C for 5 min. The supernatant (800 μl) was transferred to Millipore 5-kDa cutoff filter (UltrafreeMC-PLHCC, HMT), and the filters were centrifuged at 9100×*g*, 4 °C for 2–5 h until no liquid remained in the filter cup. The extracted sample solutions were completely evaporated and resuspended in 50 μl of Milli-Q water for metabolome analysis at HMT. Metabolome analysis was performed by Basic Scan package of HMT using capillary electrophoresis time-of-flight mass spectrometry (Human Metabolome Technologies, Inc., Tokyo, Japan)

### GAPDH activity assay

The GAPDH activity was assayed in whole cells using a commercial GAPDH activity assay kit (BioVision #680-100, Milpitas, CA, USA). Briefly, 5 × 10^5^ cells were homogenized with 100 μl of GAPDH assay buffer. Samples were kept on ice for 10 min and centrifuged at 10,000×*g*, 4^o^C for 5 min. The GAPDH activity in the supernatants was studied according to the manufacturer’s protocol. The absorbance at 450 nm was measured every 10 min for 1 h. The experiments were done in triplicates and were repeated three times.

### ECAR and OCR measurement

Extracellular acidification rate (ECAR) and oxygen consumption rate (OCR) were measured by extracellular flux (XF24) analyzer (Seahorse Bioscience) using glycolysis stress test kit (Agilent Technologies #103020-100) and cell mito stress test kit (Agilent Technologies #103015-100), respectively. Briefly, cells were seeded at 2 × 10^4^ cells per well in XF24 plates in 100 μl of culture medium and incubated for 16–20 h at 37 °C and 5% CO_2_ prior to assay. For ECAR measurement, cell medium was replaced by XF assay medium supplemented with 2 mM glutamine and incubated at the incubator without supplied CO_2_ for 1 h before the completion of probe cartridge calibration. Basal ECAR was measured in the XF assay medium without glucose, and glycolysis was measured by injecting glucose (10 mM), oligomycin (1 μM), and 2-deoxy glucose (50 mM) from XF24 reagent ports as indicated. For OCR measurement, cell medium was replaced by the 2% FBS culture medium and incubated at the incubator without CO_2_ for 1 h before the completion of probe cartridge calibration. Basal oxygen consumption rate (OCAR) was measured after injection of oligomycin (1 μM), carbonyl cyanide-4-(trifluoromethoxy) phenylhydrazone (0.5 μM), and rotenone (2 μM).

### Drug synergy analysis

For drug combination experiments, cells were treated with heptelidic acid or oligomycin for 48 h to determine the concentration that induced a 50% inhibition of cellular growth (IC50) in the MTT assay. Heptelidic acid was combined with oligomycin at a constant ratio determined by IC_50 Heptelidic Acid_/IC_50 Oligomycin_. Inhibition of cell growth by the combination of these two inhibitors was measured by MTT assay. The effects of drug combinations were evaluated with Calcusyn software (Biosoft) according to Chou–Talalay combination index method [24]. CI > 1 indicates antagonism, CI = 1 indicates additive effect, and CI < 1 indicates synergism. All experiments were carried out in triplicate.

### Xenograft animal experiments

All animal experiments were approved by Animal Care Committee of National Health Research Institutes. Advanced severe immunodeficiency (ASID) mice at 4–5 weeks were housed under standard conditions. GFP- and GFP-PRMT3-overexpressing PANC-1 cells (1 × 10^6^) were suspended in 50 μl PBS mixed with 50 μl Matrige and subcutaneously injected into the right flank of the mice. Tumor burden was monitored with digital calipers twice per week, and tumor volume was estimated using the formula (length × width^2^)/2. Three weeks after injection, mice were randomly divided into two groups to receive PBS (control) and oligomycin (0.5 mg/kg) + heptelidic acid (1 mg/kg). The number of mice per group was five. All of the mice received the drugs via tumor injection twice per week. After 1 week, tumors were harvested and tumor weight was measured.

### TUNEL assay

Apoptosis of tumor tissues was analyzed using terminal deoxynucleotidyl transferase-mediated dUTP nick end labeling (TUNEL) assay (Abcam #ab66110) according to the manufacturer’s instruction. Sections were analyzed using a Leica DMi8 microscope (Leica Microsystems, Inc.). The percentage of cell death was determined by counting the number of TUNEL-positive cells in three independent fields of different slides using ImageJ software.

### Quantification and statistical analysis

Results were shown as the Means ± SEM (*n* = 3). Differences between various experimental groups were evaluated by using a two-tailed, unpaired Student’s *t* test, and *p* value less than 0.05 was considered as statistically significant.

## Results

### PRMT3 is overexpressed in pancreatic cancer and is associated with poor clinical outcome

**Fig. 1:**
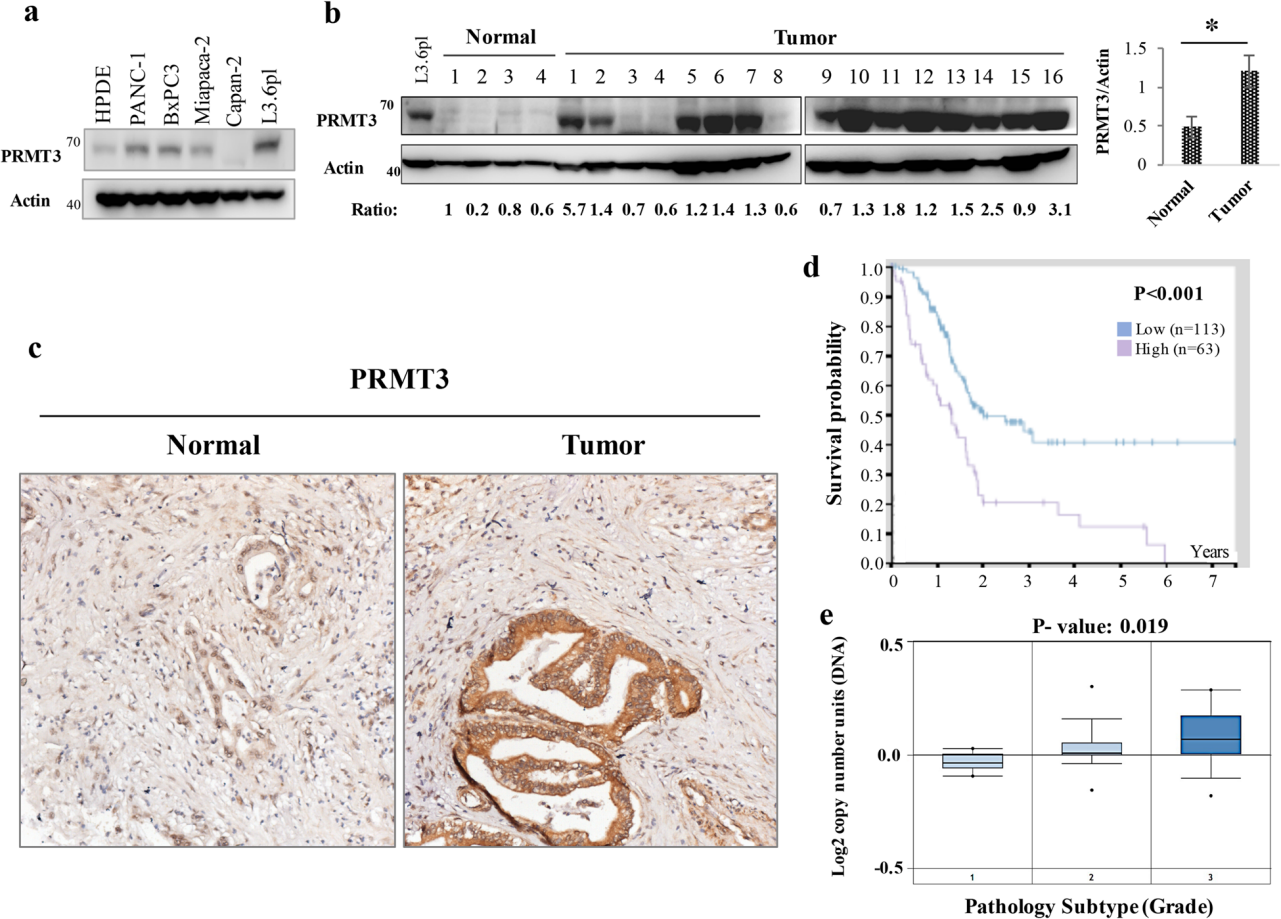
High PRMT3 expression is an unfavorable prognostic factor in pancreatic cancer patients. **a** The PRMT3 protein level in different human pancreatic cancer cell lines was determined by western blotting. **b** The PRMT3 protein levels were compared in adjacent normal and tumor parts of pancreatic tumors. The intensity of the bands was quantified by ImageJ and normalized to that of actin. The statistical graph was presented. Error bars, SEM. Normal tissues, *n* = 4; tumor tissues, *n* = 16. *p < 0.05. **c** Representative IHC staining of PRMT3 protein in human PDAC tissue. **d** The correlation between PRMT3 expression and pancreatic cancer patient survival in a TCGA cohort (https://www.proteinatlas.org/ENSG00000185238-PRMT3/pathology/ tissue/pancreatic+cancer). *p* < 0.001. **e** A box-and-whisker plot of PRMT3 gene expression in pancreatic tumors from the Oncomine dataset. The box-and-whisker 1, 2, and 3 respectively indicates pathology grade 1 (*n* = 9), grade 2 (*n* = 27), and grade 3 (*n* = 10). The correlation between PRMT3 gene expression and pathology grades was statistically significant

To verify the clinical significance of PRMT3, we compared the expression of PRMT3 in immortalized human pancreatic ductal epithelial (HPDE) cells and human pancreatic cancer cell lines and found that PRMT3 was upregulated in most of cancer cell lines (Fig. 1a). In addition, the increase of PRMT3 was detected in 69% (11/16) of the pancreatic tumor tissues investigated (Fig. 1b). Semi-quantification of the protein level by densitometry demonstrated that tumor tissues have > 2-fold increase of PRMT3 when compared to the averaged level of four adjacent normal tissues (Fig. 1b). Immunohistochemical staining showed that PRMT3 protein is mainly detected in ductal cells and its expression is significantly increased in tumor tissues (Fig. 1c). Moreover, analysis of PRMT3 expression in the 176 pancreatic cancer patients published in The Cancer Genome Atlas (TCGA) database demonstrated that high PRMT3 expression is an unfavorable prognostic factor and is associated with reduced patient survival (Fig. 1d, data derived from https://www.proteinatlas.org/ENSG00000185238-PRMT3/pathology/ tissue/pancreatic+cancer of The Human Protein Atlas) [25]. Additionally, increased PRMT3 expression is found in high-grade tumors in the Oncomine dataset (Fig. 1e). These data suggested an oncogenic role of PRMT3 in pancreatic cancer.

### GAPDH is an in vivo substrate of PRMT3

**Fig. 2:**
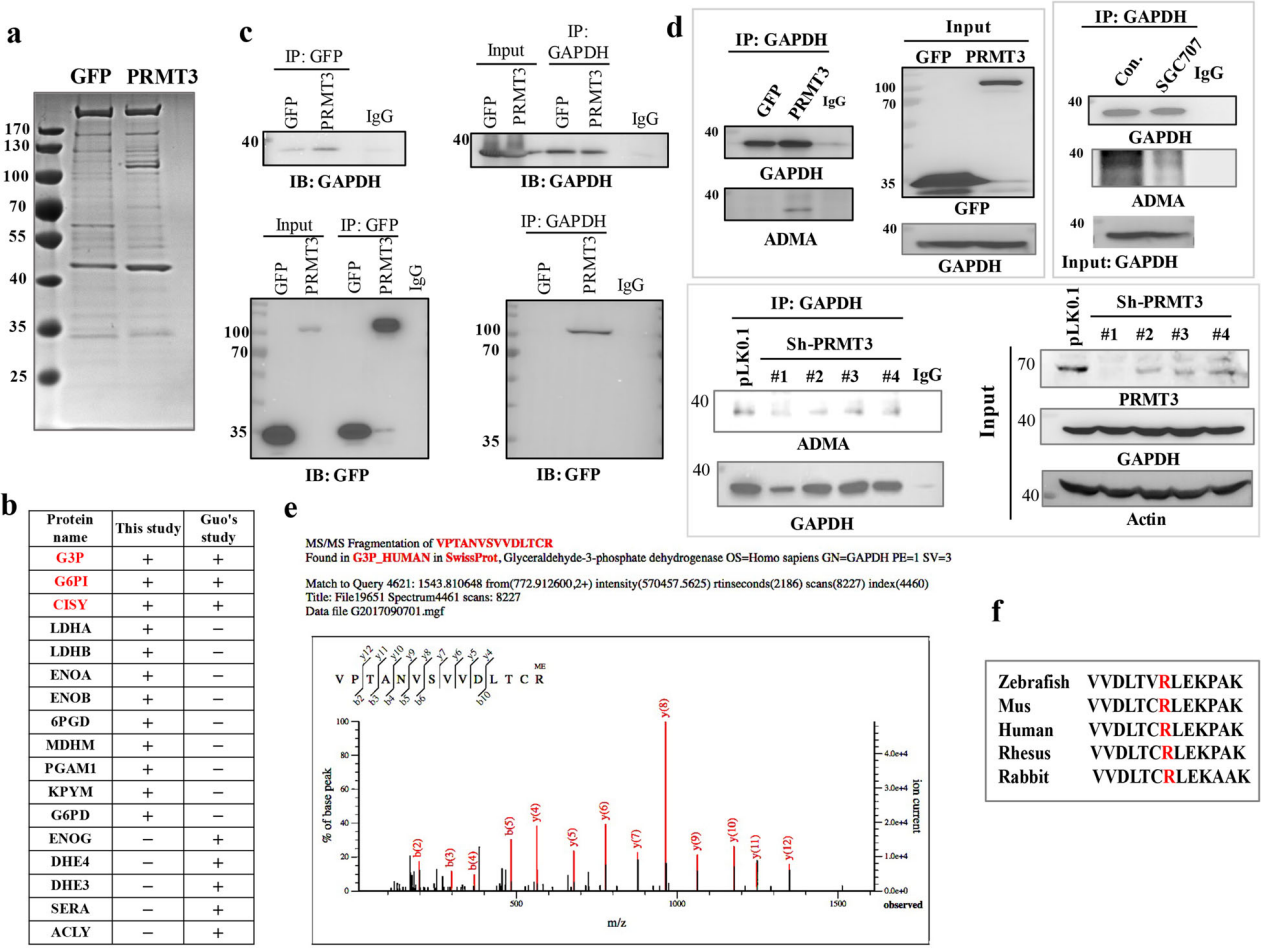
Identification of GAPDH as a novel substrate of PRMT3. **a** Coomassie blue of GFP-purified protein complexes from GFP- and GFP-PRMT3-ovexpressing PANC-1 cells. PRMT3-interacting proteins were pulled down and analyzed by mass spectrometric analysis. **b** The PRMT3-interacting metabolic enzymes identified in a previous study [19], and this study is shown and three common proteins are labeled by red color. **c** Cell lysates were collected from GFP- and GFP-PRMT3-overexpressing PANC-1 cells and subjected to immunoprecipitation using the GFP and GAPDH antibodies followed by western blotting to detect GAPDH and GFP. **d** Left upper panel, GAPDH proteins were immunoprecipitated from GFP- and GFP-PRMT3-overexpressing PANC-1 cells and subjected to western blotting to detect the level of asymmetric dimethylated arginine (ADMA). Right upper panel, L3.6pl cells were treated with SGC707 (100 μM) for 48 h. GAPDH proteins were immunoprecipitated, followed by western blotting to detect the level of ADMA. Bottom lower panel, L3.6pl cells were transfected with PRMT3-targeting shRNAs for 48 h. GAPDH proteins were immunoprecipitated and subjected to western blotting to detect the level of ADMA. **e** GAPDH proteins were purified from GFP-PRMT3-overexpressing PANC-1 cells using GAPDH antibody, and the immunoprecipitated complexes were separated by SDS-PAGE. The protein bands corresponding to GAPDH were excised and subjected to mass spectrometric analysis. **f** Alignment of the amino acid sequences around R248 of GAPDH protein in different species

To elucidate the biological function of PRMT3, we sought to identify its interacting proteins in pancreatic cancer cells. Green fluorescent protein (GFP)-tagged PRMT3 was ectopically expressed in PANC-1 cells, and the associated proteins were pulled down for proteomics analysis (Fig. 2a). A total of 293 proteins including rpS2, a confirmed substrate of PRMT3, were identified (Additional file 1: Table S1). In agreement with previous results [19], PRMT3 was found to be associated with a number of metabolic enzymes, consistent with its cytosolic location (Fig. 2b). Three interacting proteins including GAPDH, glucose-6-phosphate isomerase (G6PI), and citrate dehydrogenase (CISY) were identified in both HEK297T [19] and PANC-1 (this study) cells. We focused on GAPDH, and the interaction between PRMT3 and GAPDH was validated by immunoprecipitation/immunoblotting assay (Fig. 2c). More importantly, we detected the asymmetric dimethylarginine (ADMA) methylation of GAPDH in PANC-1 cells with ectopic expression of PRMT3 (Fig. 2d, left upper panel). The treatment of PRMT3 inhibitor SGC707 reduced the ADMA signal of GAPDH in L3.6pl cells (Fig. 2d, right upper panel). In addition, PRMT3 knockdown in L3.6pl cells decreased the ADMA signal of GAPDH (Fig. 2d, bottom lower panel). These results suggested that GAPDH could be a physiological substrate of PRMT3. Liquid chromatography coupled with tandem mass spectrometry (LC-MS/MS) identified a single methylation site at Arg248 (R248) (Fig. 2e). Sequence alignment demonstrated that this arginine residue is highly conserved in different species, indicating the methylation of this residue may have important biological significance (Fig. 2f).

### Methylation of R248 enhances the catalytic activity of GAPDH

**Fig. 3:**
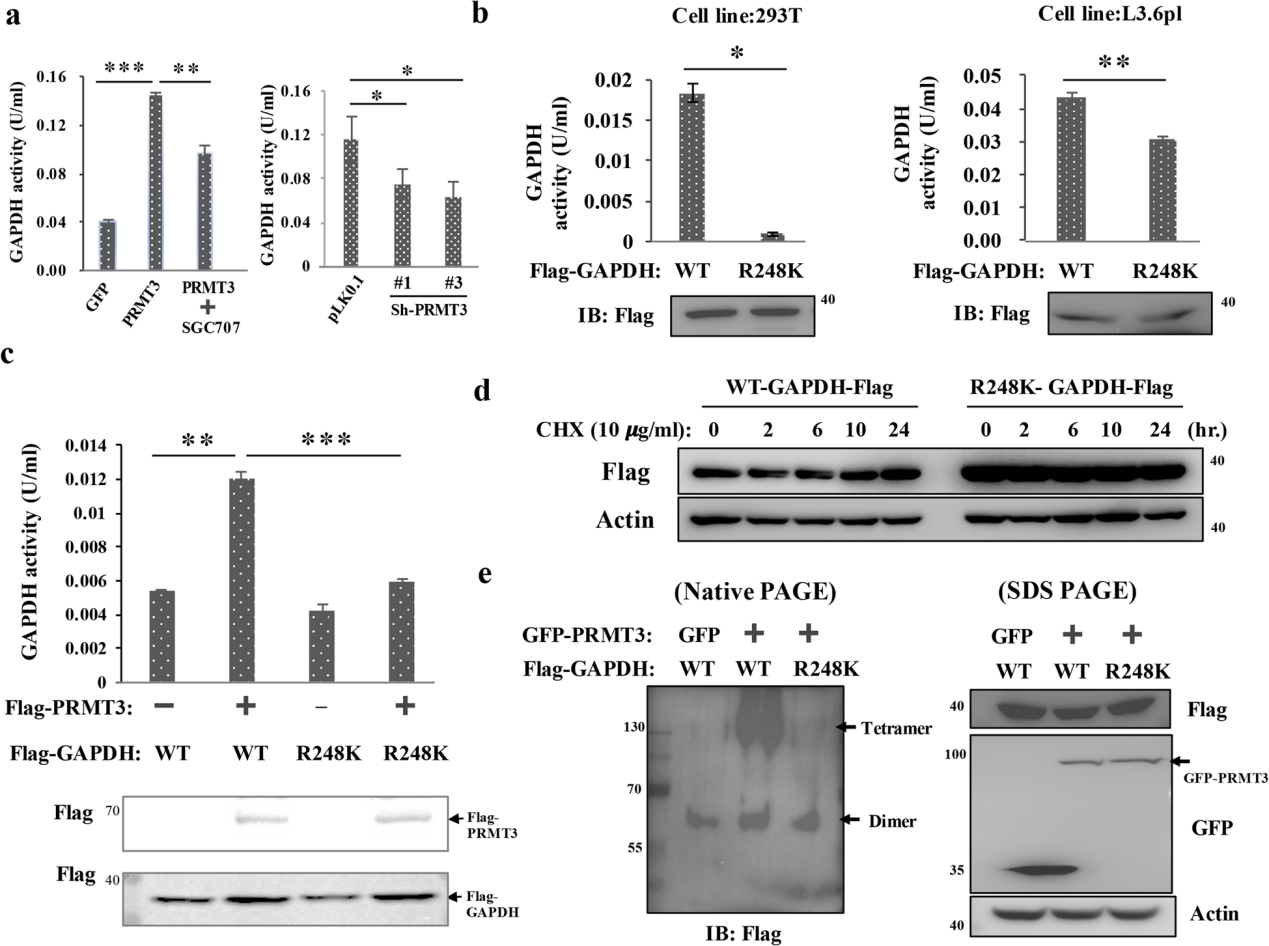
PRMT3-mediated R248 methylation increases the catalytic activity of GAPDH. **a** Left panel, the GAPDH activity of GFP-PRMT3-overexpressng PANC-1 cells treated with or without specific PRMT3 inhibitor SGC707 (100 μM) was determined by GAPDH activity kit. Right panel, the GAPDH activity of Miapaca-2 cells with or without PRMT3-targeting shRNA was determined by GAPDH activity kit. Error bars, SEM. *n* = 3. *p < 0.05, **p < 0.01, ***p < 0.001. **b** Flag-tagged wild-type GAPDH (WT) and R248K mutant expression vectors were transfected into HEK293T cells (left) and L3.6pl cells (right), respectively. After 48 h, the GAPDH activity was measured by ELISA assays and the expression levels of GAPDH were detected by western blotting. Error bars, SEM. *n* = 3. *p < 0.05, **p < 0.01. **c** HEK293T cells were co-transfected with pcDNA3, Flag-PRMT3, Flag-tagged GAPDH-WT, or R248K mutant expression vectors. After 48 h, the GAPDH activity was measured by ELISA assays and the levels of expressed proteins were detected by western blotting. Error bars, SEM. *n* = 3. **p < 0.01, ***p < 0.001. **d** Flag-tagged wild-type GAPDH (WT) and R248K mutant expression vectors were transfected into HEK293T cells. After 48 h, the cells were treated with cycloheximide (10 μg/ml) and cellular proteins were harvested at the indicated time points. The levels of Flag-tagged GAPDH were investigated by western blotting, and actin was used as an internal control. **e** Cell lysates were extracted from the indicated stable cell lines and subjected to 10% native gel electrophoresis (left) and SDS-PAGE (right), respectively. Overexpression of PRMT3 significantly enhanced the tetramer formation of wild-type GAPDH proteins but not R248-mutant GAPDH proteins

We found the GAPDH activity was increased by threefold in the PRMT3-overexpressing PANC-1 cells, and this increase was suppressed by the specific PRMT3 inhibitor SGC707. Moreover, PRMT3 knockdown in L3.6pl cells decreased GAPDH activity, suggesting PRMT3-mediated methylation of GAPDH may upregulate its catalytic activity (Fig. 3a). Mutation of Arg (R) to Lys (K) retains the positive charge of R and creates a residue that cannot be methylated by PRMT3 [26]. We generated the R248K mutant GAPDH and compared its catalytic activity with wild-type enzyme after expression in HEK293T cells. Our data showed that the activity of the R248 mutant was very low (Fig. 3b, left panel). Ectopic expression of the R248 mutant in human L3.6pl pancreatic cancer cells, which express abundant endogenous PRMT3, also decreased the total GAPDH activity in cells (Fig. 3b, right panel). Because active GAPDH is a homotetramer protein complex [27], our results suggested that the R248 mutant may interfere the assembly or activity of active tetramer. Mutation of R248 markedly reduced PRMT3-increased GAPDH activity in the HEK293T cells co-transfected with PRMT3 and GAPDH vectors (Fig. 3c). We next studied whether methylation of R248 changed the protein stability of GAPDH. Our data did not support the hypothesis because (1) the GAPDH protein levels in the control and PRMT3-overexpressing PANC-1 cells were similar (Fig. 2c) and (2) the stabilities of wild-type and R248K mutant GAPDH proteins in transfected HEK293T cells were also similar (Fig. 3d). We next tested the possibility that methylation of GAPDH at R248 by PRMT3 may promote the assembly of active tetramer. The result of native gel electrophoresis demonstrated that co-expression of PRMT3 and wild-type GAPDH increased the tetrameric form of GAPDH while co-expression of PRMT3 and GAPDH-R248K mutant did not (Fig. 3e). These data suggested that PRMT3-induced R248 methylation enhances GAPDH activity by promoting the assembly of active tetramer.

### PRMT3-mediated methylation of GAPDH promotes metabolic reprogramming

**Fig. 4:**
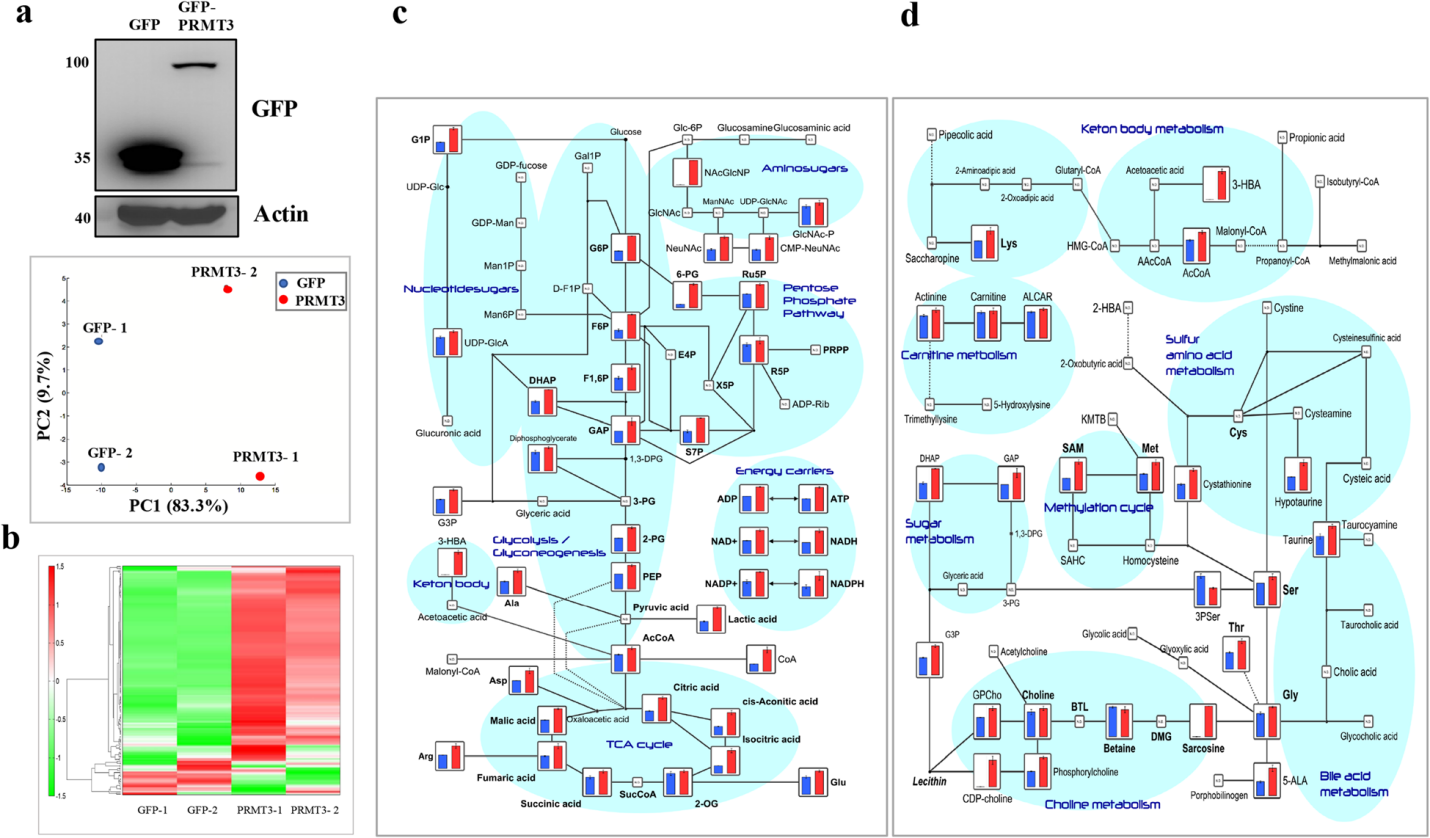
Overexpression of PRMT3 induces metabolic reprogramming. **a** Principal component analysis of the metabolites in GFP- and GFP-PRMT3-overexpressing PANC-1 cells. The expression levels of GFP and GFP-PRMT3 were detected by western blotting. **b** Hierarchical cluster analysis (HCA) of the metabolites in GFP- and GFP-PRMT3-overexpressing PANC-1 cells. The horizontal axis and vertical axis show sample names and peaks. **c** Change of the metabolites in central carbon metabolism. The bars/lines represent relative areas of each metabolite in GFP- (blue) and GFP-PRMT3 (red)-overexpressing PANC-1 cells, respectively. N.D., not detected. **d** Change of the metabolites in lipid and amino acid metabolism. The bars/lines represent relative areas of each metabolite in GFP- (blue) and GFP-PRMT3 (red)-overexpressing PANC-1 cells, respectively. N.D., not detected

**Fig. 5:**
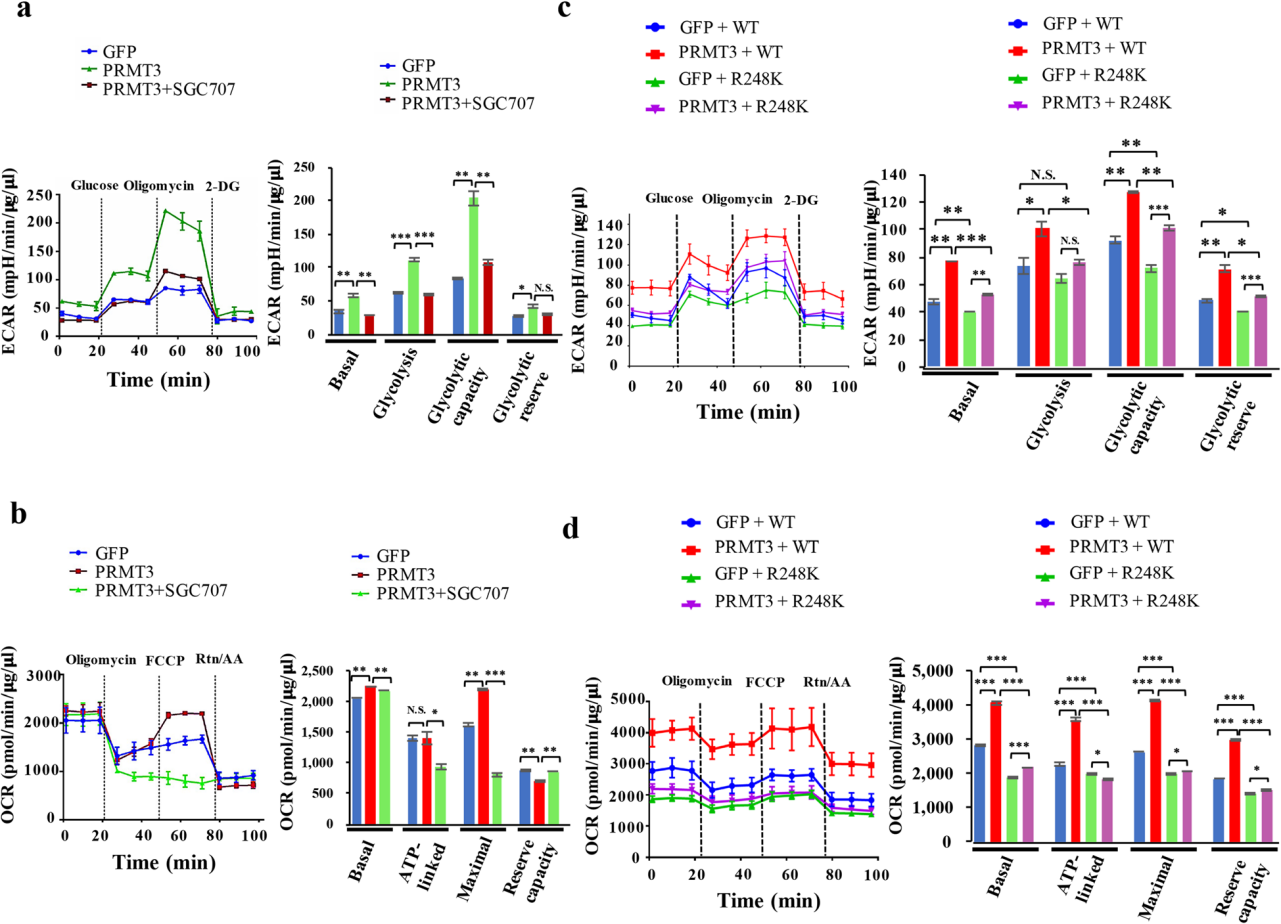
Methylation of GAPDH is important for PRMT3-induced metabolic reprogramming. **a** ECAR was measured with Seahorse XF24 Flux analyzer in GFP- and GFP-PRMT3-overexpressing PANC-1 cells with or without SGC707 treatment. Basal ECAR measurement was measured in XF assay medium without glucose, following by the addition of glucose (10 mM), oligomycin (1 μM), and 2-DG (50 mM). Error bars, SEM. *n* = 3. Column statistics of ECAR is shown in the right panel. *p < 0.05, **p < 0.01, ***p < 0.001. N.S., not significant. **b** OCR was measured in GFP- and GFP-PRMT3-overexpressing PANC-1 cells with or without SGC707 treatment. Basal OCR was measured in the Seahorse XF24 Flux analyzer. Measurements were performed by injecting oligomycin (1 μM), FCCP (0.5 μM), and rotenone (2 μM). Error bars, SEM. *n* = 3. Column statistics of OCR is shown in the right panel. *p < 0.05, **p < 0.01, ***p < 0.001. N.S., not significant. **c** ECAR was measured in PANC-1 cells with co-expression of GFP-, GFP-PRMT3, Flag-GAPDH-WT, or Flag-GAPDH-R248K mutant. Error bars, SEM. *n* = 3. Column statistics of ECAR is shown in the right panel. *p < 0.05, **p < 0.01, ***p < 0.001. N.S., not significant. **d** OCR was measured in PANC-1 cells with co-expression of GFP-, GFP-PRMT3, Flag-GAPDH-WT, or Flag-GAPDH-R248K mutant. Error bars, SEM. *n* = 3. Column statistics of OCR is shown in the right panel. *p < 0.05, ***p < 0.001

To address the biological consequence induced by PRMT3-mediated methylation of GAPDH, intracellular metabolites were analyzed by capillary electrophoresis time-of-flight mass spectrometry. We detected 174 metabolites in the control and PRMT3-overexpressing PNAC-1 cells, and principle component analysis revealed a significant difference of the metabolites in these two cell lines (Fig. 4a). Hierarchical cluster analysis also showed a dramatic alteration of intracellular metabolite levels (Fig. 4b). One of the most obviously altered pathways was the central carbon metabolism, with a significant increase of the intermediates in glycolysis and tricarboxylic acid cycle in PRMT3-overexpressing cells (Fig. 4c). In addition, the metabolism of lipids and amino acids was also upregulated, suggesting the activation of the pentose phosphate pathway (Fig. 4d). Two additional pathways affected were branched chain/aromatic amino acids and nucleotide metabolism, respectively (Additional files 2 and 3: Figures S1 and S2). Several coenzymes including NADH, NAPDH, and acetyl-coenzyme A were enriched in cells with PRMT3 overexpression (Additional file 4: Figure S3). Consistent with upregulation of glycolysis and mitochondrial respiration, the extracellular acidification rate (ECAR) and oxygen consumption rate (OCR) were both increased in PRMT3-overexpressing PANC-1 cells and were significantly inhibited by SGC707 (Fig. 5a, b). In addition to PANC-1 cells, we also tested the effect of PRMT3 inhibition on the glycolysis and mitochondrial respiration in normal HPDE cells and L3.6pl and Capan-2 pancreatic cancer cells. The treatment of SGC707 inhibited ECAR and OCR levels of L3.6pl cells more significantly than that of HPDE and Capan-2 cells which express a low level of PRMT3 protein (Additional file 5: Figure S4). To confirm that PRMT3-mediated metabolic reprogramming is dependent on GAPDH methylation, we ectopically expressed the R248K mutant in PRMT3-overexpressing PANC-1 cells and found that both ECAR and OCR were significantly suppressed (Fig. 5c, d). These data suggested that PRMT3 promotes glycolysis and mitochondrial respiration simultaneously via the methylation of GAPDH.

### Overexpression of PRMT3 sensitizes pancreatic cancer cells to GAPDH blockade

**Fig. 6:**
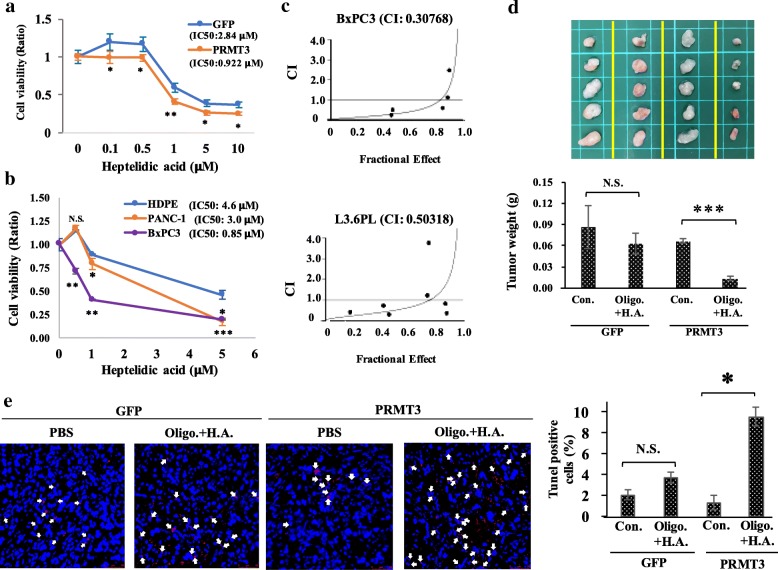
Overexpression of PRMT3 increases the sensitivity of pancreatic cancer cells to GAPDH blockage. **a** GFP- and GFP-PRMT3-ovexpressing PANC-1 cells were treated with indicated concentrations of GAPDH inhibitor, heptelidic acid, for 2 days, and the cell viability was investigated by MTT assay. Error bars, SEM. *n* = 3. *p < 0.05, **p < 0.01. **b** HPDE, PANC-1, and BxPC3 cells were treated with indicated concentrations of heptelidic acid for 2 days, and the viability was investigated by MTT assay. Error bars, SEM. *n* = 3. *p < 0.05, **p < 0.01, ***p < 0.001. N.S., not significant. **c** BxPC3 and L3.6pl cells with high endogenous expression of PRMT3 were exposed to various concentrations of heptelidic acid (between 0.05 and 2 μM) and oligomycin (between 1 and 40 μg/ml) for 48 h, and the viability was investigated by MTT assay. Combination index (CI) values were determined using the CalcuSyn software (Biosoft). CI values < 1.0 indicated a synergistic cytotoxic effect, and the CI of heptelidic acid and oligomycin in BxPC3 and L3.6pl is 0.30768 and 0.50318, respectively. **d** GFP- and GFP-PRMT3 overexpressing PANC-1 cells (1 × 10^6^) were subcutaneously injected into the right flank of ASID mice. Tumor formation was monitored with digital calipers twice per week. Three weeks after injection, mice received PBS (control) and oligomycin (Oligo., 0.5 mg/kg) + heptelidic acid (H.A., 1 mg/kg) treatment. All of the mice received the drugs via tumor injection twice per week. One week after treatment, mice were sacrificed and tumor weight was measured. Error bars, SEM. *n* = 5. N.S., not significant. ***p < 0.001. **e** Apoptosis of tumor tissues was measured by TUNEL assay, and the images were captured by a fluorescence microscope (200× magnification). White arrows indicate TUNEL-positive cells. The percentage of cell death was determined by counting the number of TUNEL-positive cells in three independent fields. Quantitative result of TUNEL assay was analyzed. Data represented mean ± SEM. Obtained from 5 mice in each group. N.S., not significant. *p < 0.05

**Fig. 7:**
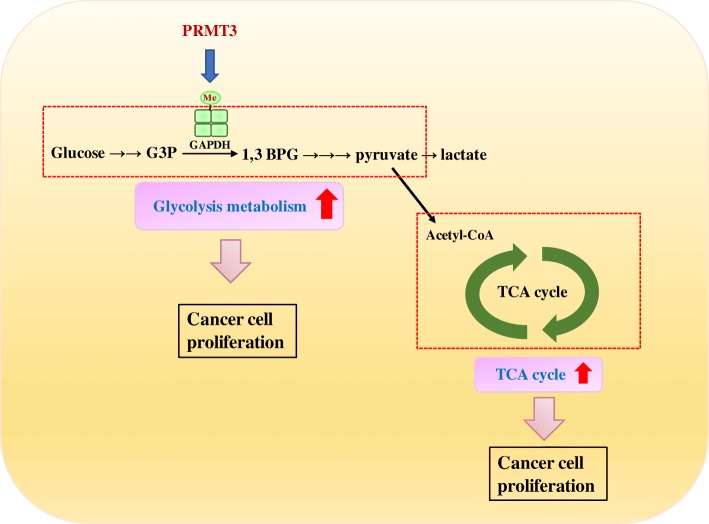
Proposed model of PRMT3-mediated metabolic reprogramming in pancreatic cancer cells. PRMT3 methylates GAPDH at arginine 248 to promote glycolysis and mitochondrial respiration simultaneously in cancer cells. Double blockade of glycolysis and mitochondrial respiration could be a novel strategy for the treatment of PRMT3-overexpressing pancreatic cancer

Because GAPDH is an important effector for PRMT3 to reprogram cellular metabolism, we hypothesized that PRMT3-overexpressing pancreatic cancer cells may be addicted to GAPDH for proliferation. Indeed, PRMT3-overexpressing PANC-1 cells were more sensitive to the GAPDH inhibitor heptelidic acid than the parental cells (Fig. 6a). Heptelidic acid also suppressed the proliferation of BxPC3 and PANC-1 pancreatic cells more significantly than that of normal HPDE cells (Fig. 6b). A unique feature of PRMT3-induced metabolic reprogramming is the simultaneous upregulation of glycolysis and mitochondrial respiration. Therefore, we tested whether the combination of oligomycin (an F0/F1 ATP synthase and mitochondrial respiration inhibitor) with heptelidic acid could elicit a more significant growth-suppressive effect. Combination index analysis confirmed these two inhibitors synergistically suppressed the proliferation of BxPC3 and L3.6pl pancreatic cancer cells (Fig. 6c). Finally, we validated the synergistic effect of oligomycin and heptelidic acid in vivo. GFP- and GFP-PRMT3-overexpressing PANC-1 cells were subcutaneously injected into the mice, and the mice were treated without or with combined drugs after tumor formation. Although we did not find a significant increase of tumor growth in the animals injecting with PRMT3-overexpressing PANC-1 cells, the percentage of Ki-67-positive cells in the tumors was increased (Additional file 6: Figure S5). Combination of oligomycin with heptelidic acid significantly suppressed tumor growth of PRMT3-overexpressing cancer cells but not that of parental PANC-1 cells (Fig. 6d). In addition, drug combination only triggered a significant increase of apoptotic cells in the PRMT3-overexpressing tumors (Fig. 6e). In another animal study, depletion of PRMT3 in Miapaca-2 pancreatic cells decreased tumor growth in vivo and increased cancer cell apoptosis in the tumor tissues (Additional file 7: Figure S6). These data suggested that PRMT3-overexpressing cancer cells are susceptive to double blockade of GAPDH and mitochondria respiration in vitro and in vivo (Fig. 7, proposed model).

## Discussion

Currently, the only cellular process confirmed to be regulated by PRMT3 in animals and plants is ribosome-mediated protein biogenesis, because the ribosomal protein rpS2 has been shown to be a methylation substrate of PRMT3 [11, 28]. Although PRMT3 has been shown to enhance hepatic lipogenesis, this effect is methylation-independent and is mediated by direct interaction between PRMT3 and liver X receptor-α, a nuclear receptor that controls the transcription of lipogenic enzymes like fatty acid synthase and acetyl-coenzyme A carboxylase [29]. In this study, we provide the first evidence that PRMT3 directly methylates GAPDH to promote glycolysis and mitochondrial respiration. The intermediates in the glycolytic pathway and tricarboxylic acid cycle are all increased in PRMT3-overexpressing cells. In addition, these cells exhibit increased ECAR and OCR, which can be reversed by ectopic expression of methylation-deficient R248K mutant GAPDH, confirming the importance of GAPDH in the regulation of cellular metabolism by PRMT3.

Posttranslational modifications (PTM) such as *S*-nitrosylation, acetylation, phosphorylation, and *O*-linked *N*-acetyl glucosamine modification of GAPDH have been demonstrated previously [30, 31]. However, little is known about arginine methylation of this glycolytic enzyme. When our study was undergoing, two studies reported that PRMT1 and PRMT4 could methylate GAPDH in cells [32, 33]. Cho et al. demonstrated that PRMT1 induces arginine methylation of GAPDH, resulting in the inhibition of GAPDH *S*-nitrosylation and nuclear localization [32]. However, no methylation site was identified in the study. Zhong et al. showed that PRMT4 methylates GAPDH at R234 and suppresses its catalytic activity to suppress glycolysis and proliferation of liver cancer cells [33]. Our results indicate that R248 is the major residue methylated by PRMT3 in vivo, and R248 methylation enhances metabolic reprogramming and cellular proliferation of pancreatic cancer cells. R248 is located at the dimer interface, which plays a critical role in the formation of active tetramer [34]. It is possible that methylation at this residue may promote tetramer assembly or stabilize active tetramer. This hypothesis is supported by our finding that mutation of R248 significantly decreases tetramer formation (Fig. 3e) and dramatically reduces GAPDH activity (Fig. 3b, c). Another important issue to be considered is the synergy or antagonism between different PTMs adjacent to R248. The Cys247 (C247) residue of GAPDH has been shown to be modified by *S*-nitrosylation, and this PTM is stimulated by oxidized low-density lipoprotein and interferon-γ [35]. Phosphorylation of Thr246 (T246) induced by protein kinase C δ under the stress of cardiac ischemia and reperfusion increases the association of GAPDH with mitochondria and inhibits GAPDH-triggered mitophagy [36]. Functional interplay between phosphorylation and arginine methylation was firstly demonstrated in the transcription factor C/EBPβ [37]. Methylation of R3 in the N-terminal transactivation domain of C/EBPβ by PRMT4 regulates the interaction of C/EBPβ with the SWI/SNF chromatin remodeling complex and alters the transcription of target genes. Interestingly, phosphorylation of T220 of C/EBPβ by mitogen-activated kinase attenuates PRMT4-mediated R3 methylation. These data suggest that phosphorylation may antagonize the effect of arginine methylation in the regulation of transcription factor activity. Whether the *S*-nitrosylation, phosphorylation, and arginine methylation at the 246–248 residues of GAPDH may occur independently, or simultaneously or consequently under various physiological or pathological circumstances, and whether the crosstalk between these PTMs may fine-tune GAPDH function to adapt extracellular alterations are important issues for further characterization.

Metabolic reprogramming is an important process for cancer cells to fit the high demand of energy requirement and supplementation of biosynthetic building blocks. Glycolysis is the metabolic pathway that converts one molecule of glucose to two molecules of pyruvate and generates two molecules of ATP and NADH per reaction. Although the efficiency of ATP production is low, the intermediates generated during the reactions could be used for synthesis of amino acids, lipids, and nucleotides to support rapid tumor growth. Therefore, many cancers switch their cellular metabolism to glycolysis under oxygen-rich conditions and the inhibition of the glycolytic pathway is considered to be a novel strategy for cancer therapy [38, 39]. However, recent studies point out that mitochondrial respiration also plays a critical role in the survival and metastasis of cancer cells [40]. In pancreatic cancer, inhibition of KRAS signaling induces extensive cancer cell death. However, a minor population of cancer cells with stemness properties may survive after oncogene ablation and those cells are highly dependent on mitochondrial respiration for survival and regrowth [41]. Similarly, chronic myeloid leukemia stem cells left after target therapy rely on mitochondrial metabolism for survival [42]. In addition, breast cancer cells may increase their invasive ability by upregulating peroxisome proliferator-activated receptor γ coactivator 1α-mediated mitochondrial biogenesis and oxidative phosphorylation [43]. An important finding of this study is the simultaneous increase of glycolysis and mitochondrial respiration in PRMT3-reprogrammed cells. This unique feature provides a molecular basis for the double blockade of these two metabolic pathways in attempts to kill PRMT3-overexpressing cancer cells. Indeed, the combination of oligomycin with heptelidic acid induces a synergistic antitumor effect in vitro and in vivo.

## Conclusion

In this study, we show that PRMT3-mediated R248 methylation of GAPDH is critical for metabolic reprogramming and cellular proliferation, and double blockade of glycolysis and mitochondrial respiration could be a novel strategy for the treatment of PRMT3-overexpressing pancreatic cancer.

## Additional files

Additional file 1: **Table S1.** PRMT3-associated proteins identified from mass spectrometry. (DOCX 36 kb)

Additional file 2: **Figure S1.** Change of the metabolites in branched chain and aromatic amino acids metabolism. The bars/lines represent relative areas of each metabolite in GFP- (blue) and GFP-PRMT3 (red)-overexpressing PANC-1 cells, respectively. N.D., not detected. (PDF 229 kb)

Additional file 3: **Figure S2.** Change of the metabolites in nucleotide metabolism. The bars/lines represent relative areas of each metabolite in GFP- (blue) and GFP-PRMT3 (red)-overexpressing PANC-1 cells, respectively. N.D., not detected. (PDF 184 kb)

Additional file 4: **Figure S3.** Change of the metabolites in metabolism of coenzymes. The bars/lines represent relative areas of each metabolite in GFP- (blue) and GFP-PRMT3 (red)-overexpressing PANC-1 cells, respectively. N.D., not detected. (PDF 492 kb)

Additional file 5: **Figure S4.** The inhibition of PRMT3 suppresses ECAR and OCR levels. (a–c) L3.6pl, HPDE, and Capan-2 cells were treated with SGC707 (100 μM) for 48 h. The ECAR and OCR levels were measured with Seahorse XF24 Flux analyzer. Error bars, SEM. *n* = 3. (PDF 3078 kb)

Additional file 6: **Figure S5.** The combination of oligomycin and heptelidic acid significantly suppresses the growth of PRMT3-overexpressing cancer cells. Cell proliferation of tumor tissues was measured by Ki67 staining, and the images were captured by a microscope. The percentage of Ki67 staining was determined by counting the number of Ki67 positive cells in three independent fields. Quantitative result of Ki67 staining was analyzed. Data represented mean ± SEM. Obtained from 5 mice in each group. *p < 0.05, ***p < 0.001. (PDF 2377 kb)

Additional file 7: **Figure S6.** Advanced severe immunodeficiency (ASID) mice were housed under standard conditions. pLK0.1- and sh-PRMT3-overexpressing Miapaca-2 cells (1 × 10^7^) were suspended in 50 μl PBS mixed with 30 μl Matrige and subcutaneously injected into the left flank of the mice. Tumor burden was monitored with digital calipers twice per week. Two weeks after injection, tumors were harvested and tumor weight was measured. Apoptosis of tumor tissues was analyzed using terminal deoxynucleotidyl transferase-mediated dUTP nick end labeling (TUNEL) assay. The percentage of cell death was determined by counting the number of TUNEL-positive cells in three independent fields of different slides using ImageJ software. The results showed that PRMT3 knockout suppressed tumor growth and increased cell apoptosis. Data represented mean ± SEM. Obtained from 5 mice in each group. (PDF 1917 kb)

## Data Availability

All data generated or analyzed during this study are included in this article and its additional files.

## Abbreviations

ADMA: Asymmetric dimethylarginine
CI: Combination index
ECAR: Extracellular acidification rate
GAPDH: Glyceraldehyde-3-phosphate dehydrogenase
HPDE: Human pancreatic ductal epithelial cells
OCR: Oxygen consumption rate
PRMTs: Protein arginine methyltransferases
SAM: *S*-Adenosyl-l-methionine
TUNEL: Terminal deoxynucleotidyl transferase-mediated dUTP nick end labeling
