# Supplementary material for: Protein arginine methyltransferase 3-induced metabolic reprogramming is a vulnerable target of pancreatic cancer
Source: J Hematol Oncol. 2019 Jul 19;12:79. doi: 10.1186/s13045-019-0769-7 (PMC6642535; doi:10.1186/s13045-019-0769-7)
Supplement: Supplementary file 1 — Table S1. PRMT3-associated proteins identified from mass spectrometry. (DOCX 36 kb) [file 13045_2019_769_MOESM1_ESM.docx]

**Supplementary tables**

**Table S1: PRMT3-associated proteins identified from mass spectrometry**

| IGKC_HUMAN | Ig kappa chain C region OS=Homo sapiens GN=IGKC PE=1 SV=1 |
| --- | --- |
| ALBU_HUMAN | Serum albumin OS=Homo sapiens GN=ALB PE=1 SV=2 |
| K2C1_HUMAN | Keratin, type II cytoskeletal 1 OS=Homo sapiens GN=KRT1 PE=1 SV=6 |
| K1C9_HUMAN | Keratin, type I cytoskeletal 9 OS=Homo sapiens GN=KRT9 PE=1 SV=3 |
| AHNK_HUMAN | Neuroblast differentiation-associated protein AHNAK OS=Homo sapiens GN=AHNAK PE=1 SV=2 |
| K1C10_HUMAN | Keratin, type I cytoskeletal 10 OS=Homo sapiens GN=KRT10 PE=1 SV=6 |
| K22E_HUMAN | Keratin, type II cytoskeletal 2 epidermal OS=Homo sapiens GN=KRT2 PE=1 SV=2 |
| ACTB_HUMAN | Actin, cytoplasmic 1 OS=Homo sapiens GN=ACTB PE=1 SV=1 |
| HS90B_HUMAN | Heat shock protein HSP 90-beta OS=Homo sapiens GN=HSP90AB1 PE=1 SV=4 |
| MYH9_HUMAN | Myosin-9 OS=Homo sapiens GN=MYH9 PE=1 SV=4 |
| TYB4_HUMAN | Thymosin beta-4 OS=Homo sapiens GN=TMSB4X PE=1 SV=2 |
| RS27A_HUMAN | Ubiquitin-40S ribosomal protein S27a OS=Homo sapiens GN=RPS27A PE=1 SV=2 |
| K2C8_HUMAN | Keratin, type II cytoskeletal 8 OS=Homo sapiens GN=KRT8 PE=1 SV=7 |
| PTMA_HUMAN | Prothymosin alpha OS=Homo sapiens GN=PTMA PE=1 SV=2 |
| CALR_HUMAN | Calreticulin OS=Homo sapiens GN=CALR PE=1 SV=1 |
| VIME_HUMAN | Vimentin OS=Homo sapiens GN=VIM PE=1 SV=4 |
| HS90A_HUMAN | Heat shock protein HSP 90-alpha OS=Homo sapiens GN=HSP90AA1 PE=1 SV=5 |
| LDHA_HUMAN | L-lactate dehydrogenase A chain OS=Homo sapiens GN=LDHA PE=1 SV=2 |
| LDHB_HUMAN | L-lactate dehydrogenase B chain OS=Homo sapiens GN=LDHB PE=1 SV=2 |
| NPM_HUMAN | Nucleophosmin OS=Homo sapiens GN=NPM1 PE=1 SV=2 |
| ENPL_HUMAN | Endoplasmin OS=Homo sapiens GN=HSP90B1 PE=1 SV=1 |
| K1C18_HUMAN | Keratin, type I cytoskeletal 18 OS=Homo sapiens GN=KRT18 PE=1 SV=2 |
| ANXA2_HUMAN | Annexin A2 OS=Homo sapiens GN=ANXA2 PE=1 SV=2 |
| ENOA_HUMAN | Alpha-enolase OS=Homo sapiens GN=ENO1 PE=1 SV=2 |
| HSP7C_HUMAN | Heat shock cognate 71 kDa protein OS=Homo sapiens GN=HSPA8 PE=1 SV=1 |
| LMNA_HUMAN | Prelamin-A/C OS=Homo sapiens GN=LMNA PE=1 SV=1 |
| EF1A1_HUMAN | Elongation factor 1-alpha 1 OS=Homo sapiens GN=EEF1A1 PE=1 SV=1 |
| EF2_HUMAN | Elongation factor 2 OS=Homo sapiens GN=EEF2 PE=1 SV=4 |
| 1433T_HUMAN | 14-3-3 protein theta OS=Homo sapiens GN=YWHAQ PE=1 SV=1 |
| FLNA_HUMAN | Filamin-A OS=Homo sapiens GN=FLNA PE=1 SV=4 |
| BASP1_HUMAN | Brain acid soluble protein 1 OS=Homo sapiens GN=BASP1 PE=1 SV=2 |
| 1433Z_HUMAN | 14-3-3 protein zeta/delta OS=Homo sapiens GN=YWHAZ PE=1 SV=1 |
| HORN_HUMAN | Hornerin OS=Homo sapiens GN=HRNR PE=1 SV=2 |
| FLNB_HUMAN | Filamin-B OS=Homo sapiens GN=FLNB PE=1 SV=2 |
| IGHG1_HUMAN | Ig gamma-1 chain C region OS=Homo sapiens GN=IGHG1 PE=1 SV=1 |
| PLEC_HUMAN | Plectin OS=Homo sapiens GN=PLEC PE=1 SV=3 |
| TBB2A_HUMAN | Tubulin beta-2A chain OS=Homo sapiens GN=TUBB2A PE=1 SV=1 |
| H4_HUMAN | Histone H4 OS=Homo sapiens GN=HIST1H4A PE=1 SV=2 |
| RS3_HUMAN | 40S ribosomal protein S3 OS=Homo sapiens GN=RPS3 PE=1 SV=2 |
| K2C1B_HUMAN | Keratin, type II cytoskeletal 1b OS=Homo sapiens GN=KRT77 PE=2 SV=3 |
| GRP78_HUMAN | 78 kDa glucose-regulated protein OS=Homo sapiens GN=HSPA5 PE=1 SV=2 |
| VINC_HUMAN | Vinculin OS=Homo sapiens GN=VCL PE=1 SV=4 |
| TYB10_HUMAN | Thymosin beta-10 OS=Homo sapiens GN=TMSB10 PE=1 SV=2 |
| K2C6B_HUMAN | Keratin, type II cytoskeletal 6B OS=Homo sapiens GN=KRT6B PE=1 SV=5 |
| K1C14_HUMAN | Keratin, type I cytoskeletal 14 OS=Homo sapiens GN=KRT14 PE=1 SV=4 |
| TRAP1_HUMAN | Heat shock protein 75 kDa, mitochondrial OS=Homo sapiens GN=TRAP1 PE=1 SV=3 |
| 1433S_HUMAN | 14-3-3 protein sigma OS=Homo sapiens GN=SFN PE=1 SV=1 |
| ENOB_HUMAN | Beta-enolase OS=Homo sapiens GN=ENO3 PE=1 SV=5 |
| EF1G_HUMAN | Elongation factor 1-gamma OS=Homo sapiens GN=EEF1G PE=1 SV=3 |
| YBOX1_HUMAN | Nuclease-sensitive element-binding protein 1 OS=Homo sapiens GN=YBX1 PE=1 SV=3 |
| ACTN4_HUMAN | Alpha-actinin-4 OS=Homo sapiens GN=ACTN4 PE=1 SV=2 |
| TPM3_HUMAN | Tropomyosin alpha-3 chain OS=Homo sapiens GN=TPM3 PE=1 SV=2 |
| TIM50_HUMAN | Mitochondrial import inner membrane translocase subunit TIM50 OS=Homo sapiens GN=TIMM50 PE=1 SV=2 |
| RS2_HUMAN | 40S ribosomal protein S2 OS=Homo sapiens GN=RPS2 PE=1 SV=2 |
| 1433B_HUMAN | 14-3-3 protein beta/alpha OS=Homo sapiens GN=YWHAB PE=1 SV=3 |
| LPPRC_HUMAN | Leucine-rich PPR motif-containing protein, mitochondrial OS=Homo sapiens GN=LRPPRC PE=1 SV=3 |
| RL3_HUMAN | 60S ribosomal protein L3 OS=Homo sapiens GN=RPL3 PE=1 SV=2 |
| HNRPK_HUMAN | Heterogeneous nuclear ribonucleoprotein K OS=Homo sapiens GN=HNRNPK PE=1 SV=1 |
| DEST_HUMAN | Destrin OS=Homo sapiens GN=DSTN PE=1 SV=3 |
| K2C5_HUMAN | Keratin, type II cytoskeletal 5 OS=Homo sapiens GN=KRT5 PE=1 SV=3 |
| K2C6A_HUMAN | Keratin, type II cytoskeletal 6A OS=Homo sapiens GN=KRT6A PE=1 SV=3 |
| ACTN1_HUMAN | Alpha-actinin-1 OS=Homo sapiens GN=ACTN1 PE=1 SV=2 |
| TBA1A_HUMAN | Tubulin alpha-1A chain OS=Homo sapiens GN=TUBA1A PE=1 SV=1 |
| LRRF1_HUMAN | Leucine-rich repeat flightless-interacting protein 1 OS=Homo sapiens GN=LRRFIP1 PE=1 SV=2 |
| FLNC_HUMAN | Filamin-C OS=Homo sapiens GN=FLNC PE=1 SV=3 |
| K1C19_HUMAN | Keratin, type I cytoskeletal 19 OS=Homo sapiens GN=KRT19 PE=1 SV=4 |
| IGHG2_HUMAN | Ig gamma-2 chain C region OS=Homo sapiens GN=IGHG2 PE=1 SV=2 |
| CH60_HUMAN | 60 kDa heat shock protein, mitochondrial OS=Homo sapiens GN=HSPD1 PE=1 SV=2 |
| ANXA5_HUMAN | Annexin A5 OS=Homo sapiens GN=ANXA5 PE=1 SV=2 |
| STMN1_HUMAN | Stathmin OS=Homo sapiens GN=STMN1 PE=1 SV=3 |
| NXN_HUMAN | Nucleoredoxin OS=Homo sapiens GN=NXN PE=1 SV=2 |
| NUCL_HUMAN | Nucleolin OS=Homo sapiens GN=NCL PE=1 SV=3 |
| TPM2_HUMAN | Tropomyosin beta chain OS=Homo sapiens GN=TPM2 PE=1 SV=1 |
| RS6_HUMAN | 40S ribosomal protein S6 OS=Homo sapiens GN=RPS6 PE=1 SV=1 |
| TFR1_HUMAN | Transferrin receptor protein 1 OS=Homo sapiens GN=TFRC PE=1 SV=2 |
| TPIS_HUMAN | Triosephosphate isomerase OS=Homo sapiens GN=TPI1 PE=1 SV=3 |
| MOES_HUMAN | Moesin OS=Homo sapiens GN=MSN PE=1 SV=3 |
| IF4A1_HUMAN | Eukaryotic initiation factor 4A-I OS=Homo sapiens GN=EIF4A1 PE=1 SV=1 |
| LAC1_HUMAN | Ig lambda-1 chain C regions OS=Homo sapiens GN=IGLC1 PE=1 SV=1 |
| K1C13_HUMAN | Keratin, type I cytoskeletal 13 OS=Homo sapiens GN=KRT13 PE=1 SV=4 |
| LA_HUMAN | Lupus La protein OS=Homo sapiens GN=SSB PE=1 SV=2 |
| H15_HUMAN | Histone H1.5 OS=Homo sapiens GN=HIST1H1B PE=1 SV=3 |
| RAB8A_HUMAN | Ras-related protein Rab-8A OS=Homo sapiens GN=RAB8A PE=1 SV=1 |
| MT1E_HUMAN | Metallothionein-1E OS=Homo sapiens GN=MT1E PE=1 SV=1 |
| RAB10_HUMAN | Ras-related protein Rab-10 OS=Homo sapiens GN=RAB10 PE=1 SV=1 |
| 1433G_HUMAN | 14-3-3 protein gamma OS=Homo sapiens GN=YWHAG PE=1 SV=2 |
| PLOD2_HUMAN | Procollagen-lysine,2-oxoglutarate 5-dioxygenase 2 OS=Homo sapiens GN=PLOD2 PE=1 SV=2 |
| PSA1_HUMAN | Proteasome subunit alpha type-1 OS=Homo sapiens GN=PSMA1 PE=1 SV=1 |
| DC1I2_HUMAN | Cytoplasmic dynein 1 intermediate chain 2 OS=Homo sapiens GN=DYNC1I2 PE=1 SV=3 |
| PAIRB_HUMAN | Plasminogen activator inhibitor 1 RNA-binding protein OS=Homo sapiens GN=SERBP1 PE=1 SV=2 |
| HS71A_HUMAN | Heat shock 70 kDa protein 1A OS=Homo sapiens GN=HSPA1A PE=1 SV=1 |
| HSP76_HUMAN | Heat shock 70 kDa protein 6 OS=Homo sapiens GN=HSPA6 PE=1 SV=2 |
| GBLP_HUMAN | Guanine nucleotide-binding protein subunit beta-2-like 1 OS=Homo sapiens GN=GNB2L1 PE=1 SV=3 |
| PDIA1_HUMAN | Protein disulfide-isomerase OS=Homo sapiens GN=P4HB PE=1 SV=3 |
| HNRPD_HUMAN | Heterogeneous nuclear ribonucleoprotein D0 OS=Homo sapiens GN=HNRNPD PE=1 SV=1 |
| PA2G4_HUMAN | Proliferation-associated protein 2G4 OS=Homo sapiens GN=PA2G4 PE=1 SV=3 |
| SRP14_HUMAN | Signal recognition particle 14 kDa protein OS=Homo sapiens GN=SRP14 PE=1 SV=2 |
| PARK7_HUMAN | Protein deglycase DJ-1 OS=Homo sapiens GN=PARK7 PE=1 SV=2 |
| STMN2_HUMAN | Stathmin-2 OS=Homo sapiens GN=STMN2 PE=1 SV=3 |
| COX17_HUMAN | Cytochrome c oxidase copper chaperone OS=Homo sapiens GN=COX17 PE=1 SV=2 |
| RS14_HUMAN | 40S ribosomal protein S14 OS=Homo sapiens GN=RPS14 PE=1 SV=3 |
| RTN4_HUMAN | Reticulon-4 OS=Homo sapiens GN=RTN4 PE=1 SV=2 |
| GCN1L_HUMAN | Translational activator GCN1 OS=Homo sapiens GN=GCN1L1 PE=1 SV=6 |
| EFHD2_HUMAN | EF-hand domain-containing protein D2 OS=Homo sapiens GN=EFHD2 PE=1 SV=1 |
| 6PGD_HUMAN | 6-phosphogluconate dehydrogenase, decarboxylating OS=Homo sapiens GN=PGD PE=1 SV=3 |
| E41L2_HUMAN | Band 4.1-like protein 2 OS=Homo sapiens GN=EPB41L2 PE=1 SV=1 |
| SUMO2_HUMAN | Small ubiquitin-related modifier 2 OS=Homo sapiens GN=SUMO2 PE=1 SV=3 |
| K2C75_HUMAN | Keratin, type II cytoskeletal 75 OS=Homo sapiens GN=KRT75 PE=1 SV=2 |
| IF2A_HUMAN | Eukaryotic translation initiation factor 2 subunit 1 OS=Homo sapiens GN=EIF2S1 PE=1 SV=3 |
| CBX3_HUMAN | Chromobox protein homolog 3 OS=Homo sapiens GN=CBX3 PE=1 SV=4 |
| IPO7_HUMAN | Importin-7 OS=Homo sapiens GN=IPO7 PE=1 SV=1 |
| HNRH1_HUMAN | Heterogeneous nuclear ribonucleoprotein H OS=Homo sapiens GN=HNRNPH1 PE=1 SV=4 |
| HN1_HUMAN | Hematological and neurological expressed 1 protein OS=Homo sapiens GN=HN1 PE=1 SV=3 |
| ITB1_HUMAN | Integrin beta-1 OS=Homo sapiens GN=ITGB1 PE=1 SV=2 |
| XRCC5_HUMAN | X-ray repair cross-complementing protein 5 OS=Homo sapiens GN=XRCC5 PE=1 SV=3 |
| CLIC4_HUMAN | Chloride intracellular channel protein 4 OS=Homo sapiens GN=CLIC4 PE=1 SV=4 |
| RS18_HUMAN | 40S ribosomal protein S18 OS=Homo sapiens GN=RPS18 PE=1 SV=3 |
| SYSC_HUMAN | Serine--tRNA ligase, cytoplasmic OS=Homo sapiens GN=SARS PE=1 SV=3 |
| 1433E_HUMAN | 14-3-3 protein epsilon OS=Homo sapiens GN=YWHAE PE=1 SV=1 |
| H2A1D_HUMAN | Histone H2A type 1-D OS=Homo sapiens GN=HIST1H2AD PE=1 SV=2 |
| RL31_HUMAN | 60S ribosomal protein L31 OS=Homo sapiens GN=RPL31 PE=1 SV=1 |
| IF4G2_HUMAN | Eukaryotic translation initiation factor 4 gamma 2 OS=Homo sapiens GN=EIF4G2 PE=1 SV=1 |
| CH10_HUMAN | 10 kDa heat shock protein, mitochondrial OS=Homo sapiens GN=HSPE1 PE=1 SV=2 |
| TCPB_HUMAN | T-complex protein 1 subunit beta OS=Homo sapiens GN=CCT2 PE=1 SV=4 |
| SYMC_HUMAN | Methionine--tRNA ligase, cytoplasmic OS=Homo sapiens GN=MARS PE=1 SV=2 |
| GRP75_HUMAN | Stress-70 protein, mitochondrial OS=Homo sapiens GN=HSPA9 PE=1 SV=2 |
| CALM_HUMAN | Calmodulin OS=Homo sapiens GN=CALM1 PE=1 SV=2 |
| PCNP_HUMAN | PEST proteolytic signal-containing nuclear protein OS=Homo sapiens GN=PCNP PE=1 SV=2 |
| IF4G1_HUMAN | Eukaryotic translation initiation factor 4 gamma 1 OS=Homo sapiens GN=EIF4G1 PE=1 SV=4 |
| RPAB3_HUMAN | DNA-directed RNA polymerases I, II, and III subunit RPABC3 OS=Homo sapiens GN=POLR2H PE=1 SV=4 |
| RS15A_HUMAN | 40S ribosomal protein S15a OS=Homo sapiens GN=RPS15A PE=1 SV=2 |
| FKBP3_HUMAN | Peptidyl-prolyl cis-trans isomerase FKBP3 OS=Homo sapiens GN=FKBP3 PE=1 SV=1 |
| ROA1_HUMAN | Heterogeneous nuclear ribonucleoprotein A1 OS=Homo sapiens GN=HNRNPA1 PE=1 SV=5 |
| TGON2_HUMAN | Trans-Golgi network integral membrane protein 2 OS=Homo sapiens GN=TGOLN2 PE=1 SV=2 |
| SAE1_HUMAN | SUMO-activating enzyme subunit 1 OS=Homo sapiens GN=SAE1 PE=1 SV=1 |
| HDGF_HUMAN | Hepatoma-derived growth factor OS=Homo sapiens GN=HDGF PE=1 SV=1 |
| HYOU1_HUMAN | Hypoxia up-regulated protein 1 OS=Homo sapiens GN=HYOU1 PE=1 SV=1 |
| RL22_HUMAN | 60S ribosomal protein L22 OS=Homo sapiens GN=RPL22 PE=1 SV=2 |
| ATLA3_HUMAN | Atlastin-3 OS=Homo sapiens GN=ATL3 PE=1 SV=1 |
| MYH10_HUMAN | Myosin-10 OS=Homo sapiens GN=MYH10 PE=1 SV=3 |
| PCBP1_HUMAN | Poly(rC)-binding protein 1 OS=Homo sapiens GN=PCBP1 PE=1 SV=2 |
| PRS7_HUMAN | 26S protease regulatory subunit 7 OS=Homo sapiens GN=PSMC2 PE=1 SV=3 |
| NDKA_HUMAN | Nucleoside diphosphate kinase A OS=Homo sapiens GN=NME1 PE=1 SV=1 |
| EIF3C_HUMAN | Eukaryotic translation initiation factor 3 subunit C OS=Homo sapiens GN=EIF3C PE=1 SV=1 |
| PROF1_HUMAN | Profilin-1 OS=Homo sapiens GN=PFN1 PE=1 SV=2 |
| CLIC1_HUMAN | Chloride intracellular channel protein 1 OS=Homo sapiens GN=CLIC1 PE=1 SV=4 |
| RLA2_HUMAN | 60S acidic ribosomal protein P2 OS=Homo sapiens GN=RPLP2 PE=1 SV=1 |
| HMGB3_HUMAN | High mobility group protein B3 OS=Homo sapiens GN=HMGB3 PE=1 SV=4 |
| AHNK2_HUMAN | Protein AHNAK2 OS=Homo sapiens GN=AHNAK2 PE=1 SV=2 |
| HS904_HUMAN | Putative heat shock protein HSP 90-alpha A4 OS=Homo sapiens GN=HSP90AA4P PE=5 SV=1 |
| MDHM_HUMAN | Malate dehydrogenase, mitochondrial OS=Homo sapiens GN=MDH2 PE=1 SV=3 |
| ACTN3_HUMAN | Alpha-actinin-3 OS=Homo sapiens GN=ACTN3 PE=1 SV=2 |
| LC7L2_HUMAN | Putative RNA-binding protein Luc7-like 2 OS=Homo sapiens GN=LUC7L2 PE=1 SV=2 |
| BIEA_HUMAN | Biliverdin reductase A OS=Homo sapiens GN=BLVRA PE=1 SV=2 |
| RS27_HUMAN | 40S ribosomal protein S27 OS=Homo sapiens GN=RPS27 PE=1 SV=3 |
| TEBP_HUMAN | Prostaglandin E synthase 3 OS=Homo sapiens GN=PTGES3 PE=1 SV=1 |
| ECHA_HUMAN | Trifunctional enzyme subunit alpha, mitochondrial OS=Homo sapiens GN=HADHA PE=1 SV=2 |
| PLIN3_HUMAN | Perilipin-3 OS=Homo sapiens GN=PLIN3 PE=1 SV=3 |
| RL28_HUMAN | 60S ribosomal protein L28 OS=Homo sapiens GN=RPL28 PE=1 SV=3 |
| RABP2_HUMAN | Cellular retinoic acid-binding protein 2 OS=Homo sapiens GN=CRABP2 PE=1 SV=2 |
| G3BP1_HUMAN | Ras GTPase-activating protein-binding protein 1 OS=Homo sapiens GN=G3BP1 PE=1 SV=1 |
| ROA2_HUMAN | Heterogeneous nuclear ribonucleoproteins A2/B1 OS=Homo sapiens GN=HNRNPA2B1 PE=1 SV=2 |
| SETLP_HUMAN | Protein SETSIP OS=Homo sapiens GN=SETSIP PE=1 SV=1 |
| PRPS1_HUMAN | Ribose-phosphate pyrophosphokinase 1 OS=Homo sapiens GN=PRPS1 PE=1 SV=2 |
| RAB6A_HUMAN | Ras-related protein Rab-6A OS=Homo sapiens GN=RAB6A PE=1 SV=3 |
| RB33B_HUMAN | Ras-related protein Rab-33B OS=Homo sapiens GN=RAB33B PE=1 SV=1 |
| RAB15_HUMAN | Ras-related protein Rab-15 OS=Homo sapiens GN=RAB15 PE=1 SV=1 |
| SNX1_HUMAN | Sorting nexin-1 OS=Homo sapiens GN=SNX1 PE=1 SV=3 |
| ODO2_HUMAN | Dihydrolipoyllysine-residue succinyltransferase component of 2-oxoglutarate dehydrogenase complex,  mitochondrial OS=Homo sapiens GN=DLST PE=1 SV=4 |
| STIP1_HUMAN | Stress-induced-phosphoprotein 1 OS=Homo sapiens GN=STIP1 PE=1 SV=1 |
| RS8_HUMAN | 40S ribosomal protein S8 OS=Homo sapiens GN=RPS8 PE=1 SV=2 |
| RL13_HUMAN | 60S ribosomal protein L13 OS=Homo sapiens GN=RPL13 PE=1 SV=4 |
| R13P3_HUMAN | Putative 60S ribosomal protein L13a protein RPL13AP3 OS=Homo sapiens GN=RPL13AP3 PE=5 SV=1 |
| TCPD_HUMAN | T-complex protein 1 subunit delta OS=Homo sapiens GN=CCT4 PE=1 SV=4 |
| RLA0L_HUMAN | 60S acidic ribosomal protein P0-like OS=Homo sapiens GN=RPLP0P6 PE=5 SV=1 |
| GGCT_HUMAN | Gamma-glutamylcyclotransferase OS=Homo sapiens GN=GGCT PE=1 SV=1 |
| MARCS_HUMAN | Myristoylated alanine-rich C-kinase substrate OS=Homo sapiens GN=MARCKS PE=1 SV=4 |
| FA49B_HUMAN | Protein FAM49B OS=Homo sapiens GN=FAM49B PE=1 SV=1 |
| RL18_HUMAN | 60S ribosomal protein L18 OS=Homo sapiens GN=RPL18 PE=1 SV=2 |
| PROF2_HUMAN | Profilin-2 OS=Homo sapiens GN=PFN2 PE=1 SV=3 |
| SYEP_HUMAN | Bifunctional glutamate/proline--tRNA ligase OS=Homo sapiens GN=EPRS PE=1 SV=5 |
| HNRPF_HUMAN | Heterogeneous nuclear ribonucleoprotein F OS=Homo sapiens GN=HNRNPF PE=1 SV=3 |
| H2B1A_HUMAN | Histone H2B type 1-A OS=Homo sapiens GN=HIST1H2BA PE=1 SV=3 |
| CALX_HUMAN | Calnexin OS=Homo sapiens GN=CANX PE=1 SV=2 |
| PSMD2_HUMAN | 26S proteasome non-ATPase regulatory subunit 2 OS=Homo sapiens GN=PSMD2 PE=1 SV=3 |
| SAHH_HUMAN | Adenosylhomocysteinase OS=Homo sapiens GN=AHCY PE=1 SV=4 |
| RS4X_HUMAN | 40S ribosomal protein S4, X isoform OS=Homo sapiens GN=RPS4X PE=1 SV=2 |
| SEPT7_HUMAN | Septin-7 OS=Homo sapiens GN=SEPT7 PE=1 SV=2 |
| PGAM1_HUMAN | Phosphoglycerate mutase 1 OS=Homo sapiens GN=PGAM1 PE=1 SV=2 |
| 2AAA_HUMAN | Serine/threonine-protein phosphatase 2A 65 kDa regulatory subunit A alpha isoform  OS=Homo sapiens GN=PPP2R1A PE=1 SV=4 |
| PDIA3_HUMAN | Protein disulfide-isomerase A3 OS=Homo sapiens GN=PDIA3 PE=1 SV=4 |
| S10AB_HUMAN | Protein S100-A11 OS=Homo sapiens GN=S100A11 PE=1 SV=2 |
| KV302_HUMAN | Ig kappa chain V-III region SIE OS=Homo sapiens PE=1 SV=1 |
| FAS_HUMAN | Fatty acid synthase OS=Homo sapiens GN=FASN PE=1 SV=3 |
| FUBP1_HUMAN | Far upstream element-binding protein 1 OS=Homo sapiens GN=FUBP1 PE=1 SV=3 |
| PLP2_HUMAN | Proteolipid protein 2 OS=Homo sapiens GN=PLP2 PE=1 SV=1 |
| ECH1_HUMAN | Delta(3,5)-Delta(2,4)-dienoyl-CoA isomerase, mitochondrial OS=Homo sapiens GN=ECH1 PE=1 SV=2 |
| NAA15_HUMAN | N-alpha-acetyltransferase 15, NatA auxiliary subunit OS=Homo sapiens GN=NAA15 PE=1 SV=1 |
| FSCN1_HUMAN | Fascin OS=Homo sapiens GN=FSCN1 PE=1 SV=3 |
| NT5D1_HUMAN | 5'-nucleotidase domain-containing protein 1 OS=Homo sapiens GN=NT5DC1 PE=1 SV=1 |
| DYHC1_HUMAN | Cytoplasmic dynein 1 heavy chain 1 OS=Homo sapiens GN=DYNC1H1 PE=1 SV=5 |
| RL18A_HUMAN | 60S ribosomal protein L18a OS=Homo sapiens GN=RPL18A PE=1 SV=2 |
| TCPH_HUMAN | T-complex protein 1 subunit eta OS=Homo sapiens GN=CCT7 PE=1 SV=2 |
| COPB_HUMAN | Coatomer subunit beta OS=Homo sapiens GN=COPB1 PE=1 SV=3 |
| HPRT_HUMAN | Hypoxanthine-guanine phosphoribosyltransferase OS=Homo sapiens GN=HPRT1 PE=1 SV=2 |
| SEPT9_HUMAN | Septin-9 OS=Homo sapiens GN=SEPT9 PE=1 SV=2 |
| GSTO1_HUMAN | Glutathione S-transferase omega-1 OS=Homo sapiens GN=GSTO1 PE=1 SV=2 |
| RL17_HUMAN | 60S ribosomal protein L17 OS=Homo sapiens GN=RPL17 PE=1 SV=3 |
| K2C79_HUMAN | Keratin, type II cytoskeletal 79 OS=Homo sapiens GN=KRT79 PE=1 SV=2 |
| BAF_HUMAN | Barrier-to-autointegration factor OS=Homo sapiens GN=BANF1 PE=1 SV=1 |
| VIGLN_HUMAN | Vigilin OS=Homo sapiens GN=HDLBP PE=1 SV=2 |
| SAP_HUMAN | Prosaposin OS=Homo sapiens GN=PSAP PE=1 SV=2 |
| PEBP1_HUMAN | Phosphatidylethanolamine-binding protein 1 OS=Homo sapiens GN=PEBP1 PE=1 SV=3 |
| NP1L1_HUMAN | Nucleosome assembly protein 1-like 1 OS=Homo sapiens GN=NAP1L1 PE=1 SV=1 |
| PSME1_HUMAN | Proteasome activator complex subunit 1 OS=Homo sapiens GN=PSME1 PE=1 SV=1 |
| DBNL_HUMAN | Drebrin-like protein OS=Homo sapiens GN=DBNL PE=1 SV=1 |
| PHP14_HUMAN | 14 kDa phosphohistidine phosphatase OS=Homo sapiens GN=PHPT1 PE=1 SV=1 |
| MYL9_HUMAN | Myosin regulatory light polypeptide 9 OS=Homo sapiens GN=MYL9 PE=1 SV=4 |
| CLCB_HUMAN | Clathrin light chain B OS=Homo sapiens GN=CLTB PE=1 SV=1 |
| GDIA_HUMAN | Rab GDP dissociation inhibitor alpha OS=Homo sapiens GN=GDI1 PE=1 SV=2 |
| COPA_HUMAN | Coatomer subunit alpha OS=Homo sapiens GN=COPA PE=1 SV=2 |
| ATPA_HUMAN | ATP synthase subunit alpha, mitochondrial OS=Homo sapiens GN=ATP5A1 PE=1 SV=1 |
| MBOA7_HUMAN | Lysophospholipid acyltransferase 7 OS=Homo sapiens GN=MBOAT7 PE=1 SV=2 |
| ENAH_HUMAN | Protein enabled homolog OS=Homo sapiens GN=ENAH PE=1 SV=2 |
| RAB7B_HUMAN | Ras-related protein Rab-7b OS=Homo sapiens GN=RAB7B PE=2 SV=1 |
| RPN2_HUMAN | Dolichyl-diphosphooligosaccharide--protein glycosyltransferase subunit 2 OS=Homo sapiens GN=RPN2 PE=1 SV=3 |
| ADPPT_HUMAN | L-aminoadipate-semialdehyde dehydrogenase-phosphopantetheinyl transferase  OS=Homo sapiens GN=AASDHPPT PE=1 SV=2 |
| PTMS_HUMAN | Parathymosin OS=Homo sapiens GN=PTMS PE=1 SV=2 |
| HNRDL_HUMAN | Heterogeneous nuclear ribonucleoprotein D-like OS=Homo sapiens GN=HNRNPDL PE=1 SV=3 |
| DCTP1_HUMAN | dCTP pyrophosphatase 1 OS=Homo sapiens GN=DCTPP1 PE=1 SV=1 |
| DENR_HUMAN | Density-regulated protein OS=Homo sapiens GN=DENR PE=1 SV=2 |
| KPYM_HUMAN | Pyruvate kinase PKM OS=Homo sapiens GN=PKM PE=1 SV=4 |
| HDGR2_HUMAN | Hepatoma-derived growth factor-related protein 2 OS=Homo sapiens GN=HDGFRP2 PE=1 SV=1 |
| PDIA6_HUMAN | Protein disulfide-isomerase A6 OS=Homo sapiens GN=PDIA6 PE=1 SV=1 |
| RL7A_HUMAN | 60S ribosomal protein L7a OS=Homo sapiens GN=RPL7A PE=1 SV=2 |
| TXD17_HUMAN | Thioredoxin domain-containing protein 17 OS=Homo sapiens GN=TXNDC17 PE=1 SV=1 |
| PFKAP_HUMAN | ATP-dependent 6-phosphofructokinase, platelet type OS=Homo sapiens GN=PFKP PE=1 SV=2 |
| G6PI_HUMAN | Glucose-6-phosphate isomerase OS=Homo sapiens GN=GPI PE=1 SV=4 |
| DEK_HUMAN | Protein DEK OS=Homo sapiens GN=DEK PE=1 SV=1 |
| PEX14_HUMAN | Peroxisomal membrane protein PEX14 OS=Homo sapiens GN=PEX14 PE=1 SV=1 |
| IMB1_HUMAN | Importin subunit beta-1 OS=Homo sapiens GN=KPNB1 PE=1 SV=2 |
| VASP_HUMAN | Vasodilator-stimulated phosphoprotein OS=Homo sapiens GN=VASP PE=1 SV=3 |
| NPC2_HUMAN | Epididymal secretory protein E1 OS=Homo sapiens GN=NPC2 PE=1 SV=1 |
| ARF5_HUMAN | ADP-ribosylation factor 5 OS=Homo sapiens GN=ARF5 PE=1 SV=2 |
| NLTP_HUMAN | Non-specific lipid-transfer protein OS=Homo sapiens GN=SCP2 PE=1 SV=2 |
| FIBG_HUMAN | Fibrinogen gamma chain OS=Homo sapiens GN=FGG PE=1 SV=3 |
| SCRN1_HUMAN | Secernin-1 OS=Homo sapiens GN=SCRN1 PE=1 SV=2 |
| RL6_HUMAN | 60S ribosomal protein L6 OS=Homo sapiens GN=RPL6 PE=1 SV=3 |
| EIF3A_HUMAN | Eukaryotic translation initiation factor 3 subunit A OS=Homo sapiens GN=EIF3A PE=1 SV=1 |
| NDUBA_HUMAN | NADH dehydrogenase [ubiquinone] 1 beta subcomplex subunit 10 OS=Homo sapiens GN=NDUFB10 PE=1 SV=3 |
| MRP_HUMAN | MARCKS-related protein OS=Homo sapiens GN=MARCKSL1 PE=1 SV=2 |
| XPO2_HUMAN | Exportin-2 OS=Homo sapiens GN=CSE1L PE=1 SV=3 |
| CKAP4_HUMAN | Cytoskeleton-associated protein 4 OS=Homo sapiens GN=CKAP4 PE=1 SV=2 |
| ERF3A_HUMAN | Eukaryotic peptide chain release factor GTP-binding subunit ERF3A OS=Homo sapiens GN=GSPT1 PE=1 SV=1 |
| CYTB_HUMAN | Cystatin-B OS=Homo sapiens GN=CSTB PE=1 SV=2 |
| CC154_HUMAN | Coiled-coil domain-containing protein 154 OS=Homo sapiens GN=CCDC154 PE=2 SV=4 |
| MOT4_HUMAN | Monocarboxylate transporter 4 OS=Homo sapiens GN=SLC16A3 PE=1 SV=1 |
| PSA6_HUMAN | Proteasome subunit alpha type-6 OS=Homo sapiens GN=PSMA6 PE=1 SV=1 |
| PRDX1_HUMAN | Peroxiredoxin-1 OS=Homo sapiens GN=PRDX1 PE=1 SV=1 |
| RL7_HUMAN | 60S ribosomal protein L7 OS=Homo sapiens GN=RPL7 PE=1 SV=1 |
| DLX4_HUMAN | Homeobox protein DLX-4 OS=Homo sapiens GN=DLX4 PE=1 SV=4 |
| SKP1_HUMAN | S-phase kinase-associated protein 1 OS=Homo sapiens GN=SKP1 PE=1 SV=2 |
| LAP2A_HUMAN | Lamina-associated polypeptide 2, isoform alpha OS=Homo sapiens GN=TMPO PE=1 SV=2 |
| CE350_HUMAN | Centrosome-associated protein 350 OS=Homo sapiens GN=CEP350 PE=1 SV=1 |
| RS15_HUMAN | 40S ribosomal protein S15 OS=Homo sapiens GN=RPS15 PE=1 SV=2 |
| BRD1_HUMAN | Bromodomain-containing protein 1 OS=Homo sapiens GN=BRD1 PE=1 SV=1 |
| GANAB_HUMAN | Neutral alpha-glucosidase AB OS=Homo sapiens GN=GANAB PE=1 SV=3 |
| G6PD_HUMAN | Glucose-6-phosphate 1-dehydrogenase OS=Homo sapiens GN=G6PD PE=1 SV=4 |
| G3P_HUMAN | Glyceraldehyde-3-phosphate dehydrogenase OS=Homo sapiens GN=GAPDH PE=1 SV=3 |
| PIMT_HUMAN | Protein-L-isoaspartate(D-aspartate) O-methyltransferase OS=Homo sapiens GN=PCMT1 PE=1 SV=4 |
| 5NT3B_HUMAN | 7-methylguanosine phosphate-specific 5'-nucleotidase OS=Homo sapiens GN=NT5C3B PE=1 SV=4 |
| PUS7L_HUMAN | Pseudouridylate synthase 7 homolog-like protein OS=Homo sapiens GN=PUS7L PE=1 SV=1 |
| CDK13_HUMAN | Cyclin-dependent kinase 13 OS=Homo sapiens GN=CDK13 PE=1 SV=2 |
| AT2A2_HUMAN | Sarcoplasmic/endoplasmic reticulum calcium ATPase 2 OS=Homo sapiens GN=ATP2A2 PE=1 SV=1 |
| RAB5C_HUMAN | Ras-related protein Rab-5C OS=Homo sapiens GN=RAB5C PE=1 SV=2 |
| DNJA1_HUMAN | DnaJ homolog subfamily A member 1 OS=Homo sapiens GN=DNAJA1 PE=1 SV=2 |
| CF163_HUMAN | Uncharacterized protein C6orf163 OS=Homo sapiens GN=C6orf163 PE=4 SV=2 |
| CDC42_HUMAN | Cell division control protein 42 homolog OS=Homo sapiens GN=CDC42 PE=1 SV=2 |
| RS3A_HUMAN | 40S ribosomal protein S3a OS=Homo sapiens GN=RPS3A PE=1 SV=2 |
| HBA_HUMAN | Hemoglobin subunit alpha OS=Homo sapiens GN=HBA1 PE=1 SV=2 |
| RL30_HUMAN | 60S ribosomal protein L30 OS=Homo sapiens GN=RPL30 PE=1 SV=2 |
| TES_HUMAN | Testin OS=Homo sapiens GN=TES PE=1 SV=1 |
| PPAL_HUMAN | Lysosomal acid phosphatase OS=Homo sapiens GN=ACP2 PE=1 SV=3 |
| GCYB2_HUMAN | Guanylate cyclase soluble subunit beta-2 OS=Homo sapiens GN=GUCY1B2 PE=2 SV=2 |
| CISY_HUMAN | Citrate synthase, mitochondrial OS=Homo sapiens GN=CS PE=1 SV=2 |
| FGD4_HUMAN | FYVE, RhoGEF and PH domain-containing protein 4 OS=Homo sapiens GN=FGD4 PE=1 SV=2 |
| PUF60_HUMAN | Poly(U)-binding-splicing factor PUF60 OS=Homo sapiens GN=PUF60 PE=1 SV=1 |
| EZRI_HUMAN | Ezrin OS=Homo sapiens GN=EZR PE=1 SV=4 |
| RO52_HUMAN | E3 ubiquitin-protein ligase TRIM21 OS=Homo sapiens GN=TRIM21 PE=1 SV=1 |
| EMAL4_HUMAN | Echinoderm microtubule-associated protein-like 4 OS=Homo sapiens GN=EML4 PE=1 SV=3 |
| ROA3_HUMAN | Heterogeneous nuclear ribonucleoprotein A3 OS=Homo sapiens GN=HNRNPA3 PE=1 SV=2 |
| NONO_HUMAN | Non-POU domain-containing octamer-binding protein OS=Homo sapiens GN=NONO PE=1 SV=4 |
| HTSF1_HUMAN | HIV Tat-specific factor 1 OS=Homo sapiens GN=HTATSF1 PE=1 SV=1 |
