# Supplementary figures and images for: Protein arginine methyltransferase 3-induced metabolic reprogramming is a vulnerable target of pancreatic cancer
Source: J Hematol Oncol. 2019 Jul 19;12:79. doi: 10.1186/s13045-019-0769-7 (PMC6642535; doi:10.1186/s13045-019-0769-7)

# Supplementary data

Figure S1

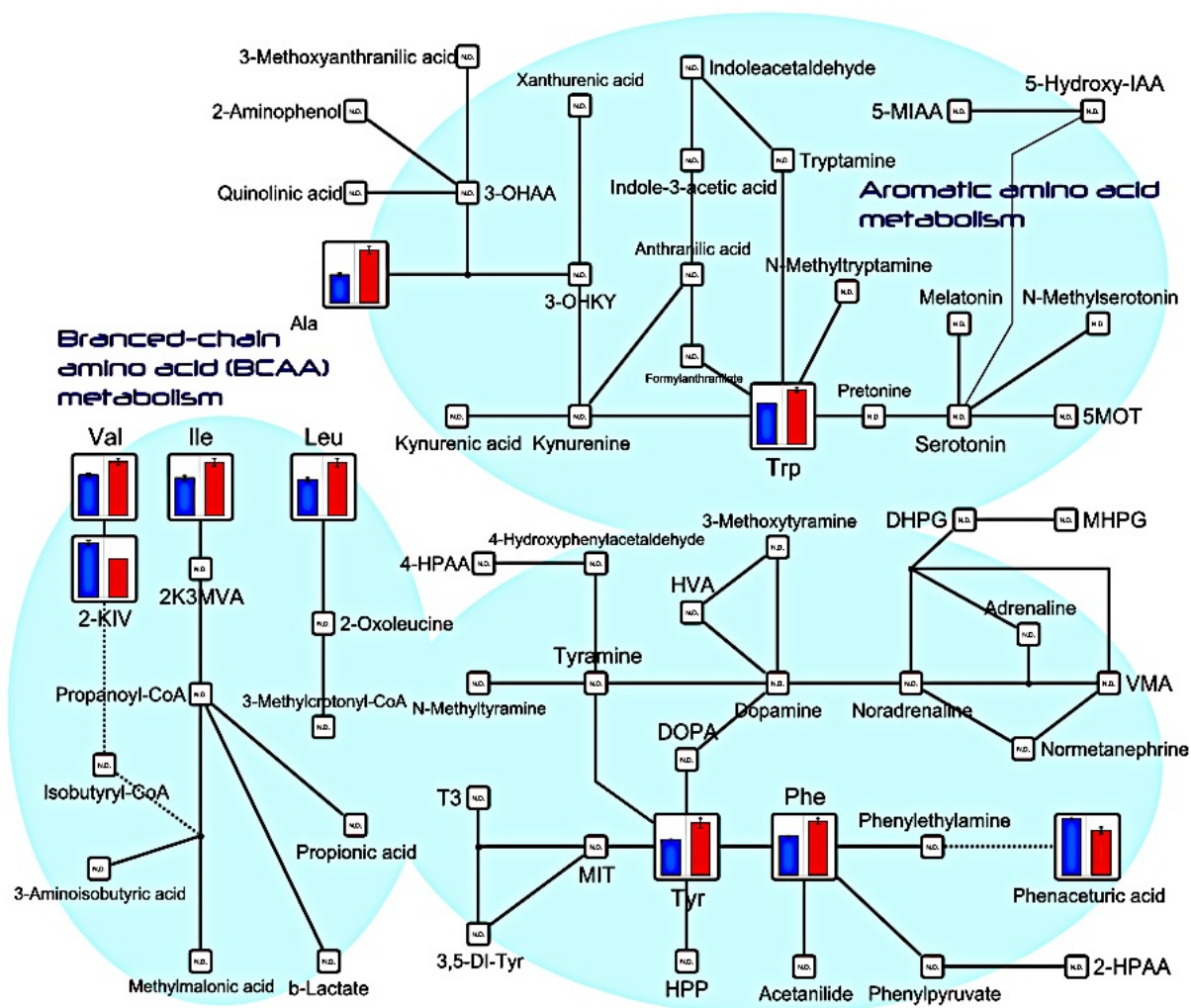

Supplement: Supplementary file 2 — Figure S1. Change of the metabolites in branched chain and aromatic amino acids metabolism. The bars/lines represent relative areas of each metabolite in GFP- (blue) and GFP-PRMT3 (red)-overexpressing PANC-1 cells, respectively. N.D., not detected. (PDF 229 kb) [file 13045_2019_769_MOESM2_ESM.pdf]

# Figure S2

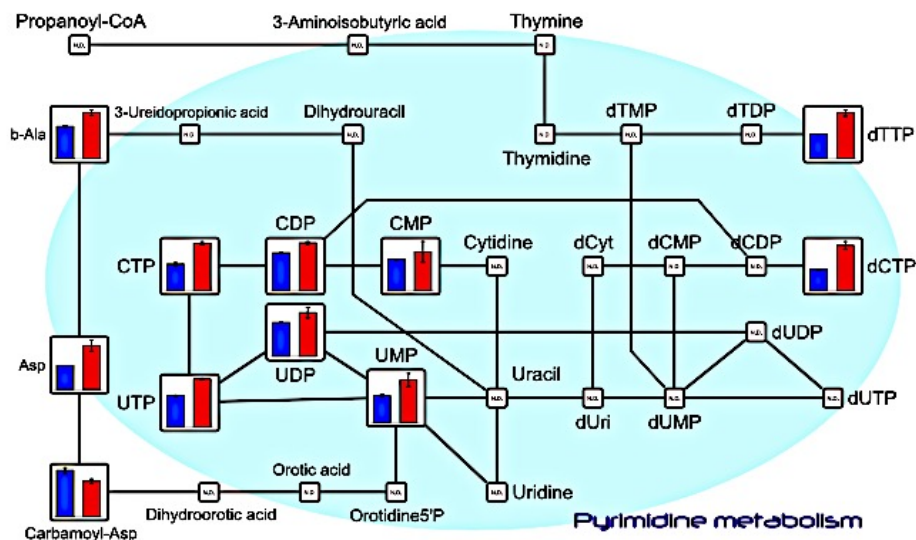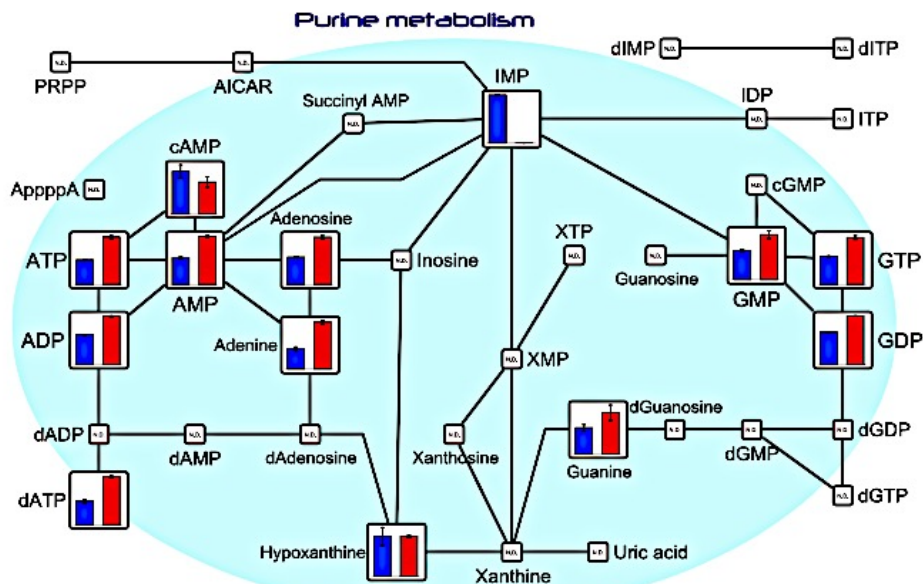

Supplement: Supplementary file 3 — Figure S2. Change of the metabolites in nucleotide metabolism. The bars/lines represent relative areas of each metabolite in GFP- (blue) and GFP-PRMT3 (red)-overexpressing PANC-1 cells, respectively. N.D., not detected. (PDF 184 kb) [file 13045_2019_769_MOESM3_ESM.pdf]

# Figure S4

**a**

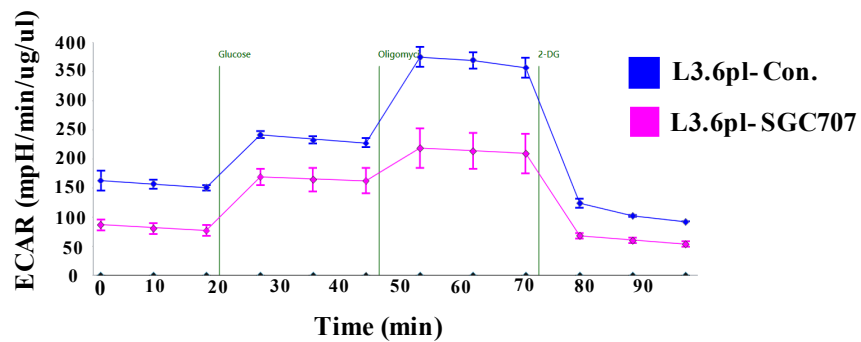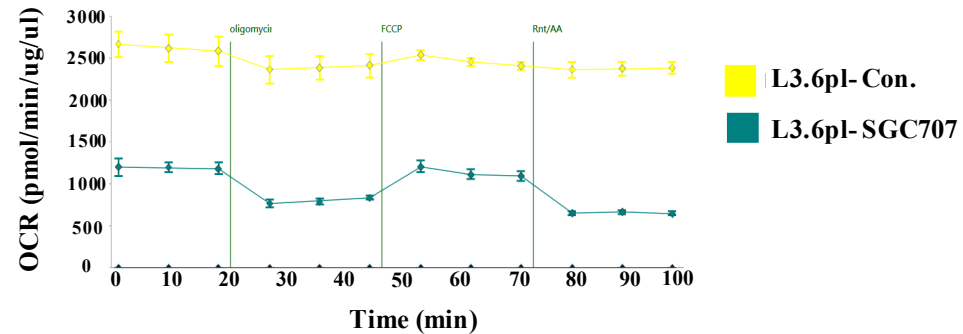

**b**

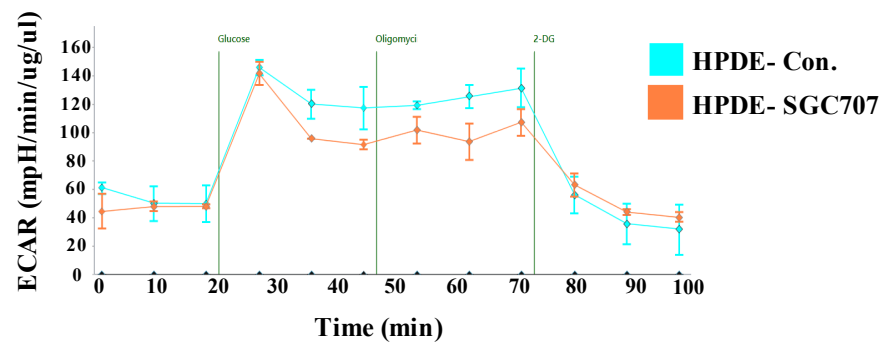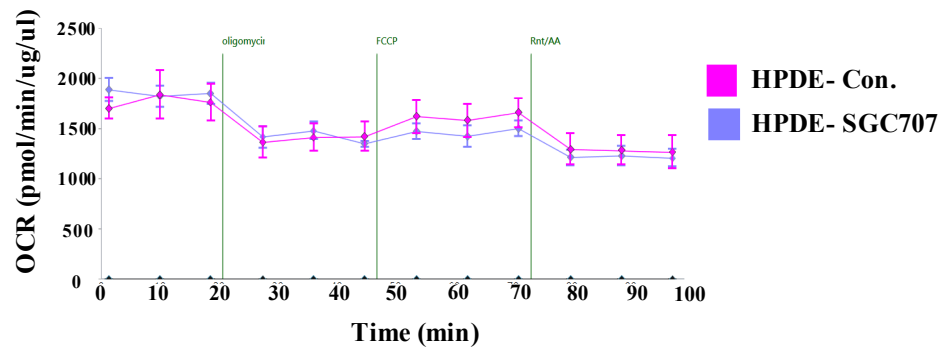

**c**

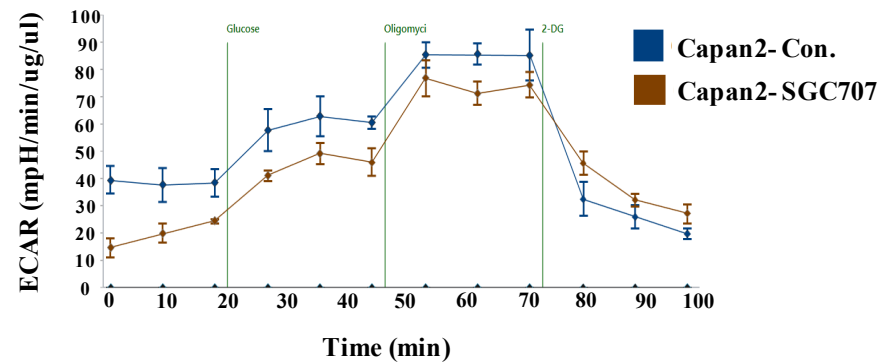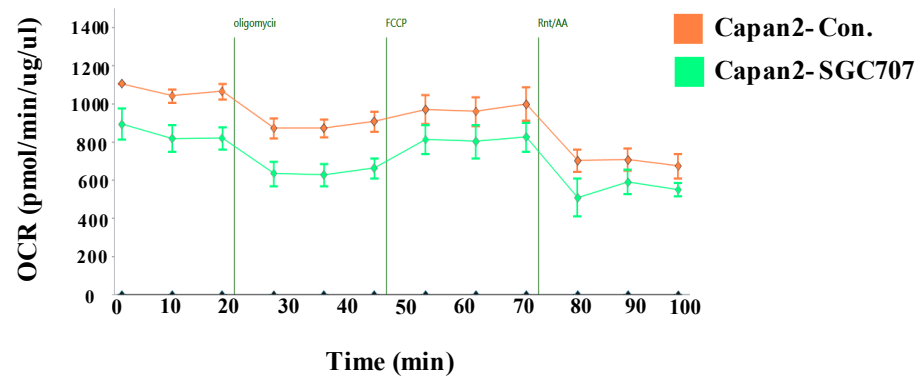

Supplement: Supplementary file 5 — Figure S4. The inhibition of PRMT3 suppresses ECAR and OCR levels. (a–c) L3.6pl, HPDE, and Capan-2 cells were treated with SGC707 (100 μM) for 48 h. The ECAR and OCR levels were measured with Seahorse XF24 Flux analyzer. Error bars, SEM. n = 3. (PDF 3078 kb) [file 13045_2019_769_MOESM5_ESM.pdf]

**GFP**

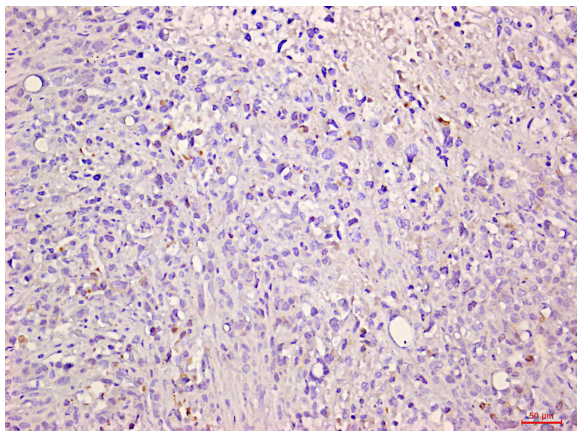

**GFP – Oligo.+H.A.**

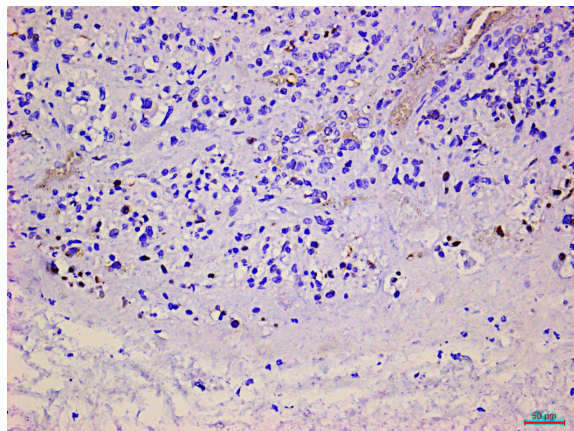

**PRMT3**

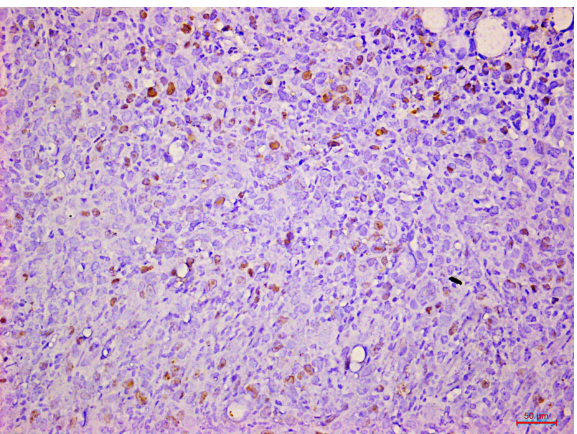

**PRMT3 – Oligo.+H.A.**

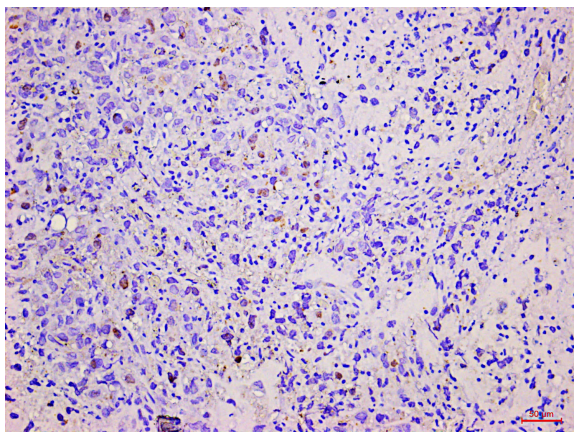

**Ki67 staining**

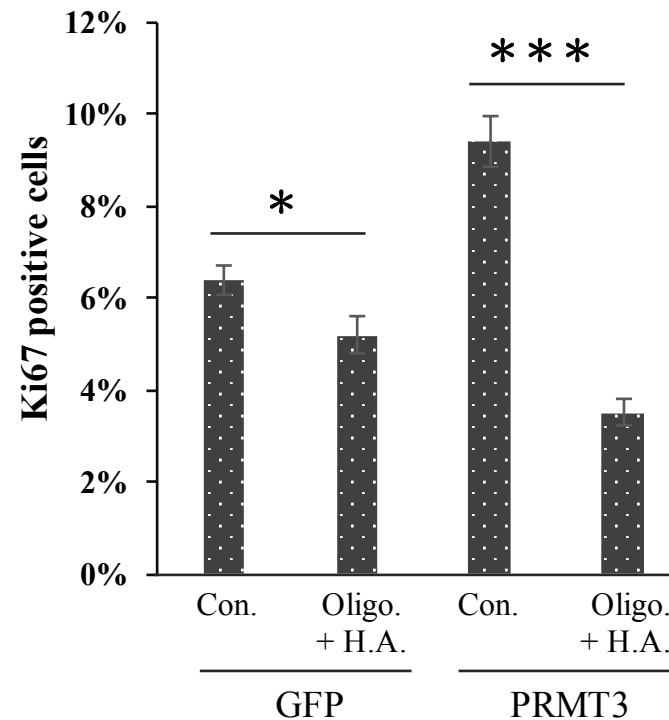

Supplement: Supplementary file 6 — Figure S5. The combination of oligomycin and heptelidic acid significantly suppresses the growth of PRMT3-overexpressing cancer cells. Cell proliferation of tumor tissues was measured by Ki67 staining, and the images were captured by a microscope. The percentage of Ki67 staining was determined by counting the number of Ki67 positive cells in three independent fields. Quantitative result of Ki67 staining was analyzed. Data represented mean ± SEM. Obtained from 5 mice in each group. *p < 0.05, ***p < 0.001. (PDF 2377 kb) [file 13045_2019_769_MOESM6_ESM.pdf]

**Figure S6**

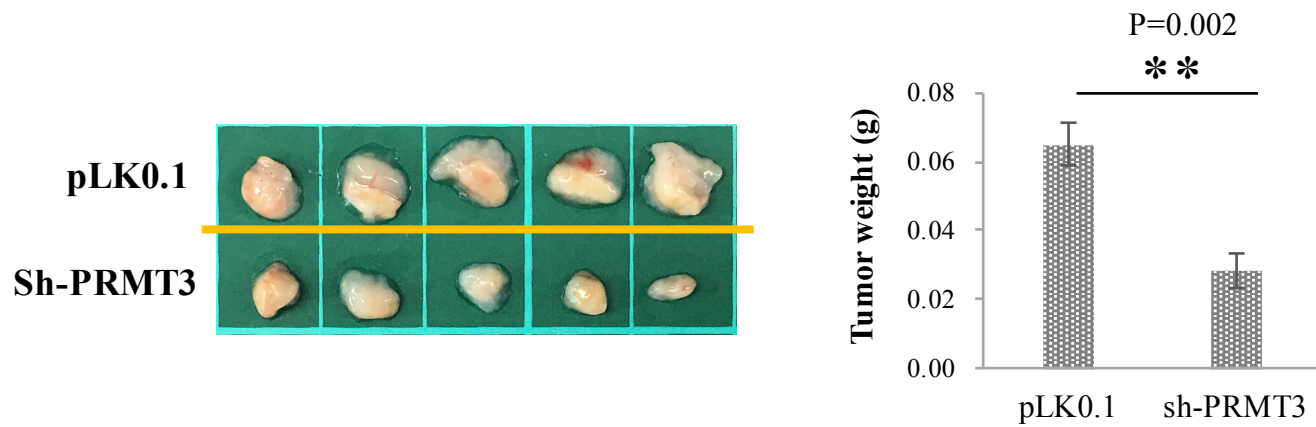

**pLK0.1**

**Sh-PRMT3**

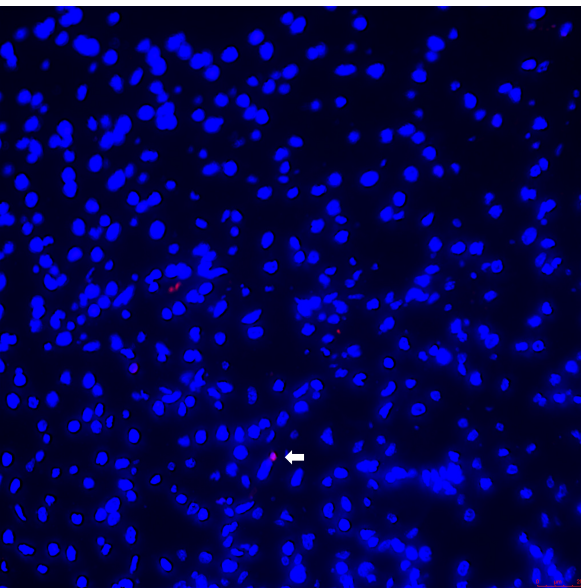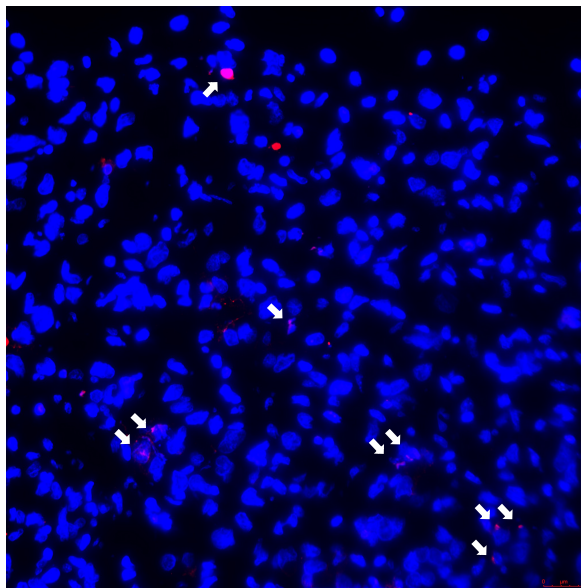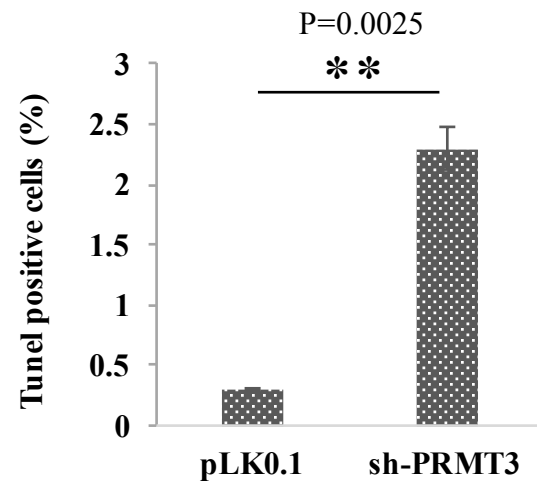

Supplement: Supplementary file 7 — Figure S6. Advanced severe immunodeficiency (ASID) mice were housed under standard conditions. pLK0.1- and sh-PRMT3-overexpressing Miapaca-2 cells (1 × 10^7^) were suspended in 50 μl PBS mixed with 30 μl Matrige and subcutaneously injected into the left flank of the mice. Tumor burden was monitored with digital calipers twice per week. Two weeks after injection, tumors were harvested and tumor weight was measured. Apoptosis of tumor tissues was analyzed using terminal deoxynucleotidyl transferase-mediated dUTP nick end labeling (TUNEL) assay. The percentage of cell death was determined by counting the number of TUNEL-positive cells in three independent fields of different slides using ImageJ software. The results showed that PRMT3 knockout suppressed tumor growth and increased cell apoptosis. Data represented mean ± SEM. Obtained from 5 mice in each group. (PDF 1917 kb) [file 13045_2019_769_MOESM7_ESM.pdf]
