# Supplementary material for: Protein arginine methyltransferase 3-induced metabolic reprogramming is a vulnerable target of pancreatic cancer
Source: J Hematol Oncol. 2019 Jul 19;12:79. doi: 10.1186/s13045-019-0769-7 (PMC6642535; doi:10.1186/s13045-019-0769-7)
Supplement: Supplementary file 4 — Figure S3. Change of the metabolites in metabolism of coenzymes. The bars/lines represent relative areas of each metabolite in GFP- (blue) and GFP-PRMT3 (red)-overexpressing PANC-1 cells, respectively. N.D., not detected. (PDF 492 kb) [file 13045_2019_769_MOESM4_ESM.pdf]

Figure S3

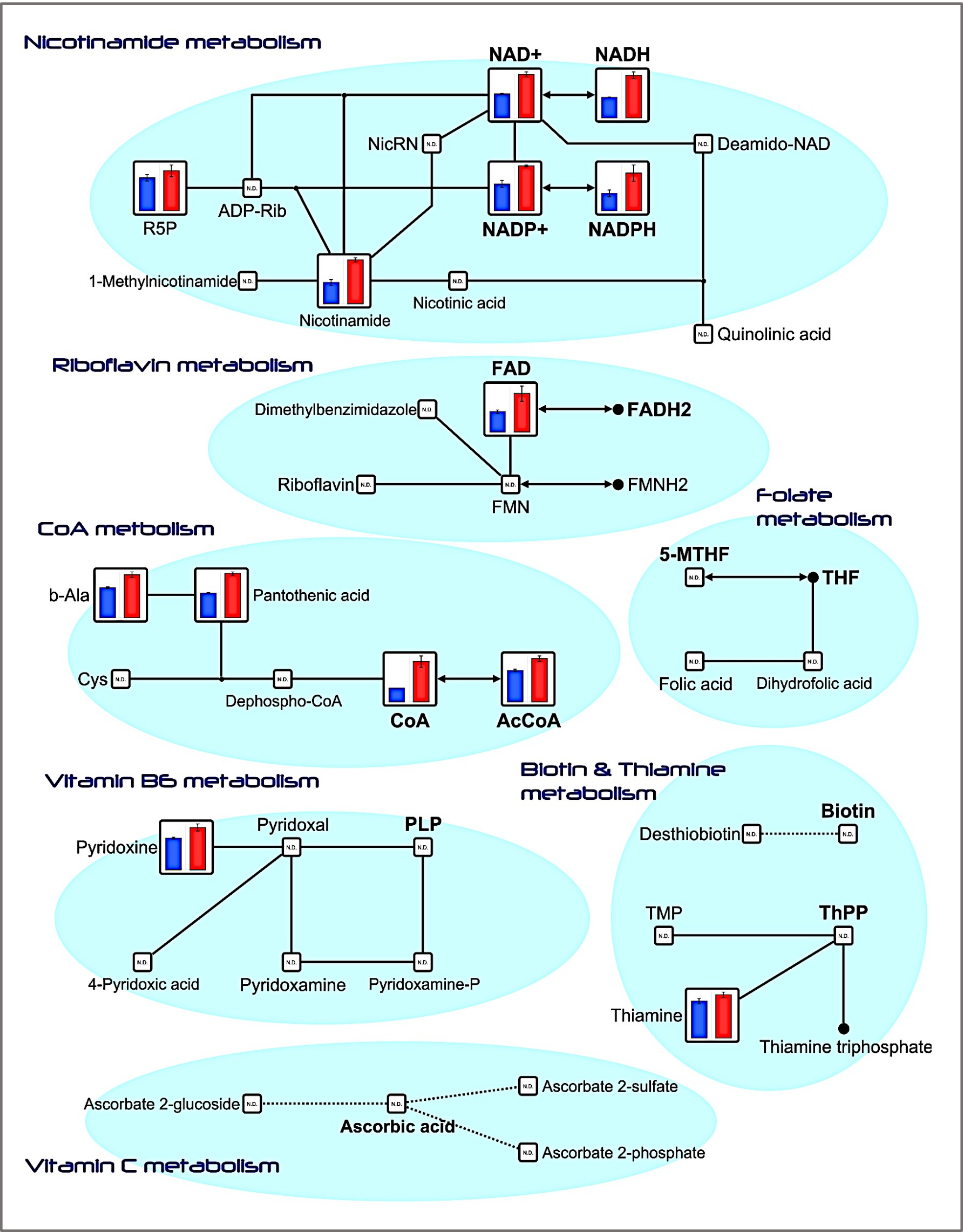
Nicotinamide metabolism
NAD+
NADH
NicRN
Deamido-NAD
R5P
ADP-Rib
NADP+
NADPH
1-Methylnicotinamide
Nicotinamide
Nicotinic acid
Quinolinic acid
Riboflavin metabolism
FAD
Dimethylbenzimidazole
FADH2
Riboflavin
FMN
FMNH2
Folate metabolism
5-MTHF
THF
Folic acid
Dihydrofolic acid
CoA metbolism
b-Ala
Pantothenic acid
Cys
Dephospho-CoA
CoA
AcCoA
Vitamin B6 metabolism
Pyridoxine
Pyridoxal
PLP
4-Pyridoxic acid
Pyridoxamine
Pyridoxamine-P
Biotin & Thiamine metabolism
Biotin
Desthiobiotin
TMP
ThPP
Thiamine
Thiamine triphosphate
Ascorbate 2-glucoside
Ascorbic acid
Ascorbate 2-sulfate
Ascorbate 2-phosphate
Vitamin C metabolism
